# Self-powered intelligence for personalized healthcare

**DOI:** 10.1093/nsr/nwag317

**Published:** 2026-05-27

**Authors:** Yeyun Cai, Ziyue Kong, Pengfei Jin, Xiangyang Wang, Ruizhe Hou, Ning Ding, Hailing Fu, Eric M Yeatman, Chengkuo Lee, Fang Deng, Jie Chen

**Affiliations:** National Key Lab of Autonomous Intelligent Unmanned Systems, Beijing Institute of Technology, Beijing 100081, China; Beijing Key Laboratory of Lightweight Intelligent System, Beijing Institute of Technology, Beijing 100081, China; School of AI, Beijing Institute of Technology, Beijing 100081, China; National Key Lab of Autonomous Intelligent Unmanned Systems, Beijing Institute of Technology, Beijing 100081, China; National Key Lab of Autonomous Intelligent Unmanned Systems, Beijing Institute of Technology, Beijing 100081, China; National Key Lab of Autonomous Intelligent Unmanned Systems, Beijing Institute of Technology, Beijing 100081, China; National Key Lab of Autonomous Intelligent Unmanned Systems, Beijing Institute of Technology, Beijing 100081, China; National Key Lab of Autonomous Intelligent Unmanned Systems, Beijing Institute of Technology, Beijing 100081, China; National Key Lab of Autonomous Intelligent Unmanned Systems, Beijing Institute of Technology, Beijing 100081, China; Beijing Key Laboratory of Lightweight Intelligent System, Beijing Institute of Technology, Beijing 100081, China; School of AI, Beijing Institute of Technology, Beijing 100081, China; College of Science and Engineering, University of Glasgow, Glasgow G12 8QQ, UK; Department of Electrical and Electronic Engineering, Imperial College London, London SW7 2AZ, UK; Department of Electrical and Computer Engineering, National University of Singapore, Singapore 117583, Singapore; National Key Lab of Autonomous Intelligent Unmanned Systems, Beijing Institute of Technology, Beijing 100081, China; Beijing Key Laboratory of Lightweight Intelligent System, Beijing Institute of Technology, Beijing 100081, China; School of AI, Beijing Institute of Technology, Beijing 100081, China; National Key Lab of Autonomous Intelligent Unmanned Systems, Beijing Institute of Technology, Beijing 100081, China; Harbin Institute of Technology, Harbin 150001, China

**Keywords:** wearable/implantable devices, personalized healthcare, lightweight intelligence, flexible and smart electronics, energy harvesting

## Abstract

Personalized healthcare is crucial for transforming medical practice, offering more precise, efficient, and patient-centerd care. It not only improves individual health outcomes but also enhances the overall efficiency of healthcare systems. Wearable and implantable devices have emerged as key technologies in this transformation, offering unprecedented opportunities for real-time physiological monitoring, early disease detection, and personalized health intervention. In parallel, the rapid advancement of artificial intelligence (AI) has further accelerated the integration of data-driven intelligence into healthcare systems, making interdisciplinary research at the intersection of AI, flexible electronics, and biomedical engineering increasingly important. However, the long-term deployment of intelligent wearable and implantable healthcare systems remains fundamentally constrained by power sustainability, mechanical flexibility, and the energy cost of on-device data processing. This review focuses on self-powered intelligence as an emerging paradigm for personalized healthcare, enabling continuous, long-term, and autonomous health monitoring beyond the limitations of battery-powered systems. By integrating flexible and smart electronics with low-power AI, self-powered intelligence provide a viable pathway toward intelligent, on-body and in-body healthcare platforms capable of real-time analysis and personalized health management.

## Teaser

This review highlights self-powered intelligence as a promising strategy for enabling sustainable, AI-driven wearable and implantable systems for continuous and personalized healthcare.

## INTRODUCTION

Medical care is a fundamental pillars of human well-being, playing a critical role in extending life expectancy and improving quality of life [1]. Despite remarkable advances in medical technologies, contemporary healthcare systems continue to face persistent challenges, including rising costs, inefficient resource utilization, and limited capability to deliver truly personalized care. Conventional healthcare models remain largely reactive, focusing on disease treatment rather than prevention, and are fundamentally constrained by the lack of continuous, long-term, and high-quality physiological data. In response to these challenges, the development of personalized healthcare has emerged as a promising paradigm aimed at shifting medical practice toward continuous monitoring, early diagnosis, and individualized intervention [2]. Enabled by wearable and implantable devices, personalized healthcare seeks to capture real-time physiological information and support tailored health management strategies that adapt to individual conditions [3,4]. In this work, personalization is defined in a hierarchical and system-level manner rather than being limited to per-user model adaptation. It encompasses population-level modeling, subgroup-level adaptation, and individual-level customization based on physiological characteristics and longitudinal data. Such personalization is typically enabled through distributed intelligence across cloud, edge, and device layers, where large-scale models are maintained in the cloud and lightweight modules are deployed on-device for real-time inference under resource constraints. This framework allows scalable yet individualized healthcare in self-powered systems. Although existing wearable and implantable devices can already achieve measurement performance comparable to regulated medical instruments, their widespread and long-term deployment remains limited by energy sustainability and insufficient on-device intelligence [5]. Most existing wearable/implantable devices rely on batteries with limited lifespans, increasing maintenance burden and user inconvenience. In particular, implantable devices such as pacemakers require battery replacement through secondary surgeries, and battery failure poses a life-threatening risk to patients. Moreover, many wearable and implantable platforms function primarily as data acquisition units, relying on external devices for data analysis and decision-making, which restricts their autonomy and responsiveness.

**Figure 1.**
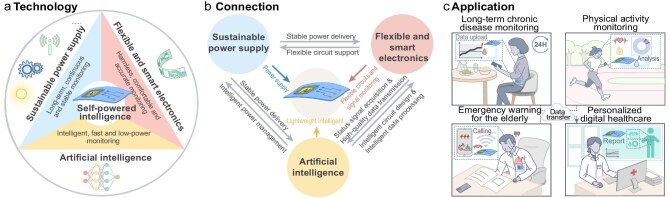
Personalized healthcare enabled by self-powered intelligence. (a) Self-powered intelligence consists of self-sustainable power supply, flexible and smart electronics, and AI. (b) The interactions among three modules within self-powered intelligence. (c) Typical applications of self-powered intelligence include chronic disease monitoring, emergency disease prediction, and sports rehabilitation monitoring.

To address these limitations, self-powered intelligence has emerged as an interdisciplinary approach that integrates energy autonomy, flexible electronics, and artificial intelligence (AI) into a unified system-level framework, aiming to enable long-term, autonomous, and intelligent healthcare operation under realistic physiological and environmental conditions. Such integration enables autonomous, continuous, and closed-loop healthcare systems, which are difficult to achieve with isolated technologies. By shifting from component-level optimization to system-level co-design, self-powered intelligence offers significant advantages in long-term operation, reduced user intervention, and enhanced adaptability under realistic physiological conditions. As illustrated in Fig. 1a, self-powered intelligence comprises three tightly coupled components: a self-sustainable power supply, flexible and smart electronics, and AI. The self-sustainable power supply (energy harvesters) converts ambient energy (such as solar, thermal, or kinetic energy) into electrical energy to support continuous operation battery recharging or replacement [6,7]. Flexible and smart electronics provide mechanical compliance and biointegration, enabling energy harvesters, sensors, and circuit management modules to conform seamlessly to the human body or internal organs [8,9]. AI further transforms these systems from passive sensing platforms into intelligent healthcare agents by enabling adaptive power management, real-time data interpretation, and decision-support capabilities [10–12].

As further illustrated in Fig. 1b, these three components are interconnected through the coordinated flow of energy, data, and feedback, forming a closed-loop system. Energy harvested from the environment powers the electronics and sensing modules, which acquire and transmit physiological signals for AI-based analysis. In turn, artificial intelligence enables adaptive feedback to dynamically optimize sensing strategies and power management. Through this synergistic and bidirectional interaction, self-powered intelligence establishes a closed-loop framework [13,14]. This integrated architecture provides unique advantages over conventional systems by enabling energy-autonomous sensing, real-time data processing, and adaptive decision-making within a single platform, which is particularly valuable for long-term and personalized healthcare applications. Representative application scenarios enabled by this framework are shown in Fig. 1c, highlighting its potential for scalable personalized healthcare and society-level health management. Typical applications of self-powered intelligence include, but are not limited to, chronic disease monitoring, emergency disease prediction, and sports rehabilitation monitoring. The collected data can be used for doctor diagnosis in personalized digital healthcare.

In this review, the focus is placed on self-powered intelligence for personal healthcare, with an exploration of the state-of-the-art advancements in self-sustainable power supply, flexible and smart electronics, and AI. While previous reviews have primarily focused on individual components such as self-powered devices or flexible electronics, this review emphasizes a system-level perspective. Emphasis is given to miniaturized, self-powered, flexible intelligent systems designed for personalized healthcare, including energy harvesters, sensors, data processing units, and novel non-Von Neumann computing architectures. The review further highlights the comprehensive role of AI in enhancing device design efficiency, power management, and data processing within the frame of self-powered intelligence. Finally, the current challenges and future directions for self-powered intelligence in personalized healthcare are summarized and discussed.

## SELF-SUSTAINABLE POWER SUPPLY

Energy autonomy is a fundamental prerequisite for the long-term deployment of intelligent wearable and implantable systems in personalized healthcare. By harvesting energy directly from the surrounding environment or the human body, self-powered intelligence aims to enable long-term energy-autonomous or energy-neutral operation under realistic conditions, thereby overcoming the limitations associated with battery replacement and periodic recharging [15], which are particularly restrictive in clinical and implantable applications. In this context, ‘self-powered’ does not necessarily imply strictly battery-free operation at all times, but rather emphasizes the ability of a system to sustainably support sensing, computation, and communication through continuous energy harvesting, often with the assistance of micro-scale energy storage units to buffer intermittent supply. In recent years, substantial progress has been made in energy harvesting technologies, and their technical maturity has been continuing to improve. However, most devices marketed as self-powered remain partially dependent on batteries, with harvested energy typically supporting only sensing components while backend processing and communication functions still rely on external power sources [16]. Most of the developed energy harvesters are still not ready to independently support long-term operation (for example, $> $1 week) of typical wearable/implantable smart devices [17]. This gap highlights the ongoing challenge of achieving fully integrated energy autonomy at the system level. Considering the unique requirements in healthcare applications, energy harvesting must therefore be evaluated not only by power output performance, but also by its compatibility with system-level constraints such as volume, placement location, and physiological conditions. In this section, self-sustainable power supply strategies for self-powered intelligence are reviewed from three perspectives: the principles of energy harvesting technologies, analysis of energy harvesting performance, and the possible placement of energy harvesters on the human body.

### Energy harvesting technology

Based on the categories of available energy, this subsection will primarily introduce kinetic, solar, heat, and radio-frequency energy harvesting transduction mechanism. Chemical and biomass energy conversion approaches are also available, but they predominantly rely on material-specific innovations and have been comprehensively reviewed elsewhere [3,17], thus they are not reviewed in this paper.

Kinetic energy harvesting mainly includes electromagnetic energy harvesters, piezoelectric energy harvesters (PEHs), and triboelectric nanogenerators (TENGs). Electromagnetic energy harvesters utilize magnets and coils to convert mechanical motion into electrical energy via electrical damping, typically necessitating large-scale mechanical movement or vibration [18]. Although electromagnetic energy harvesters generally exhibits high power density, the use of magnets poses challenges for miniaturization and thin-film applications [18]. In contrast, self-powered intelligence demands compact size, lightweight design and high flexibility to adapt to the dynamic human body.

**Figure 2.**
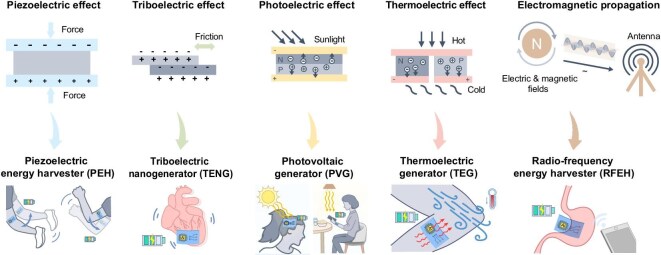
The principles of five energy harvesting technologies based on ambient/human energy and their typical examples applied on the human body.

PEHs use functional materials such as polyvinylidene fluoride to generate voltage when subjected to mechanical stress [19] (Fig. 2). It is noted that piezoelectricity arises from non-centrosymmetric crystal structures, and while certain ferroelectric materials also exhibit piezoelectric behavior, the two are not equivalent material classes but are distinguished by the presence of spontaneous polarization in ferroelectrics. In addition to single-material PEHs, piezoelectric composites have been widely explored, in which piezoelectric fillers are embedded in flexible polymer matrices. These composites offer improved mechanical compliance, durability, and energy conversion efficiency, making them particularly suitable for wearable and implantable self-powered devices [20,21]. TENGs work through triboelectrification and electrostatic induction [22] (Fig. 2). Owing to their complementary electromechanical characteristics, PEHs and TENGs are often co-designed to simultaneously harvest energy and monitor body or organ motion [23]. PEHs generally exhibit high-frequency characteristics and have limitations for low-frequency motion. Extensive efforts have been devoted to optimizing geometric configurations, incorporating composite materials, and introducing nonlinear mechanisms with AI-enabled methods to reduce the operating frequency of PEHs, thereby improving their compatibility with low-frequency human motion [24]. Numerous studies have already demonstrated the use of PEHs to provide power for wearable/implantable devices such as transdermal drug delivery systems and pacemakers [25]. On the other hand, TENGs are normally designed to harness low-frequency mechanical energy ($< $4 $\mathrm{Hz }$ [26]). TENG has been utilized for self-powered sensing in applications such as human motion rehabilitation monitoring [27], joint condition monitoring [23], and heart health monitoring [28]. Despite these advances, both PEHs and TENGs face challenges in power density, operational stability, and long-term durability due to the limited available space on the human body.

Photovoltaic generators (PVGs) convert sunlight into electrical energy with high power conversion efficiency (Fig. 2). Recent studies have reported efficiencies of $\sim$26% for advanced photovoltaic devices [29,30]. Further advances have pushed the efficiency of single-junction silicon solar cells beyond 27% [31], while multijunction photovoltaic devices can achieve efficiencies exceeding 40% and even approach $\sim$47% under concentrated illumination [32]. However, such high-efficiency systems typically rely on rigid architectures, complex fabrication processes, and high-cost materials, which limit their applicability in wearable and implantable healthcare systems requiring flexibility, lightweight design, and mechanical compliance. Emerging photovoltaics, including organic, dye-sensitized, and perovskite solar cells, are advancing rapidly but remain less mature than crystalline silicon technologies in terms of long-term stability and large-scale manufacturability. Both laboratory and industrial efforts are focused on improving their stability and optimizing manufacturing processes [33]. Flexible perovskite PVGs have been used to power wearable systems for continuous biochemical monitoring, including glucose, sodium ions, and pH [34]. Moreover, progress in all-perovskite tandem photovoltaics has recently pushed the efficiency of polycrystalline thin-film devices beyond 30% for the first time [35]. Despite significant progress, the reliability, durability, scalability, and manufacturing processes of these emerging materials still require further enhancement and validation [36]. Beyond material innovations, body-worn PVGs can leverage their reduced cost sensitivity to explore high-efficiency architectures, including multiple-bandgap and tandem structures. Furthermore, optimization for artificial indoor light spectra has become increasingly relevant, since many wearable devices operate predominantly in indoor or mixed-light environments. Nevertheless, due to the inherent dependence on light availability, PVGs must account for the position and intensity of the incident light during operation, which restricts their practicality for implantable devices and those worn beneath clothing.

Thermoelectric generators (TEGs) produce electricity via the temperature difference across the semiconductor [37] (Fig. 2). The temperature difference between the human body and ambient air provides a natural thermal gradient for TEG, making it a promising solution in healthcare applications. Significant progress has been made in thermoelectric materials study, including Si and SiGe-based materials, metals such as Cu and Ni, polymers, and chalcogenides, as well as in the fabrication of flexible and micro-scale devices [38]. Studies in thermoelectric technology continue to explore materials with a high figure of merit (ZT), a dimensionless parameter determined by the Seebeck coefficient, electrical conductivity, and thermal conductivity, to achieve higher thermoelectric conversion efficiency. A key challenge is the low temperature gradient between the skin and environment, which limits achievable power density [39]. Complementary to TEGs, pyroelectric energy harvesters, typically based on ferroelectric materials, generate electricity from temporal temperature fluctuations rather than spatial gradients, offering an alternative route for harvesting dynamic thermal variations associated with body motion or environmental changes. While still emerging, pyroelectric approaches broaden the range of usable thermal resources [40].

Although the human body continuously generates mechanical motion and metabolic heat, the harvestable fraction of this energy is fundamentally limited by human physiology. A resting adult dissipates roughly 100 $\mathrm{W}$ of metabolic power, increasing to several hundred watts during physical activity, almost all of which eventually converts to heat. Wearable and implantable kinetic and thermal energy harvesters typically operate in the microwatt-to-milliwatt range, therefore interacting with only a negligible fraction of the body’s total energy budget. These physiological considerations contextualize the practical power limits of body-based energy harvesting.

Radio-frequency energy harvesters (RFEHs) convert ambient or transmitted electromagnetic waves into usable electrical power through antenna reception, rectification, and power conditioning [15] (Fig. 2). Near-field inductive and resonant magnetic coupling systems, while differing from far-field RFEH in operating distance and coupling mechanisms, share the same electromagnetic foundations and collectively form the broader landscape of wireless power transfer. RFEHs have attracted considerable interest for powering wearable and implantable devices, including orthodontic force sensors and micro-tube pacemakers, due to its compact form factor and ability to deliver power without direct physical contact [41]. However, radio-frequency power transfer must comply with strict safety constraints. Electromagnetic attenuation in biological tissues significantly reduces transmission efficiency, often requiring higher transmitted power, while exposure limits governed by specific absorption rate (SAR) impose constraints on allowable field strength, with regulatory limits such as $\sim$1.6 $\mathrm{W/kg}$ averaged over 1 g of tissue (FCC/IEEE) or $\sim$2 $\mathrm{W/kg}$ averaged over 10 g of tissue (ICNIRP/European standard) often used as design ceilings in biomedical contexts to limit tissue heating and ensure safe operation [42,43]. In practice, allowable SAR limits significantly influence the design trade‑off between transmitted power, operating frequency, and device form factor, especially in implantable or epidermal systems where prolonged exposure and thermal safety are critical. As a result, non-radiative resonant systems and directional radiation techniques are preferred to improve efficiency while maintaining safety. Designing compact, efficient, and orientation-insensitive RFEHs under these constraints remains a major engineering priority [44].

A single energy source struggles to handle the complex and dynamic operating environment of the human body, impacting the stability and long-term reliability of the power supply. Therefore, multi-source energy harvesting technologies are emerging as well to achieve self-powered intelligence. Common hybrid energy harvesting systems typically rely on the simple stacking of different individual harvesters, such as the combination of PEH and TENG [45], or the integration of PVG and TEG [46], which may sometimes sacrifice the advantages of each individual harvester and even lead to a reduction in overall performance. Ideally, hybrid systems should transcend simple component integration to achieve synergistic enhancement. This can be realized by discovering novel multi-effect energy harvesting mechanisms (such as the tribovoltaic effect [47] and piezo-phototronic effect [48]), utilizing shared electrodes, implementing co-optimized mechanical architectures, and employing unified power management circuits. Magnetoelectric (ME) energy harvesters represent a sophisticated paradigm of such integration. By exploiting the intrinsic coupling between magnetostrictive and piezoelectric phases, they effectively transduce magnetic or mechanical stimuli into high-density electrical energy. These devices offer the dual advantages of extreme compactness and robust wireless power transfer (WPT) capabilities, making them uniquely suited for deep-tissue implantable sensors and remote physiological monitoring. Recent advancements have validated the feasibility of ME systems for driving low-power biomedical electronics, underscoring their potential in next-generation autonomous healthcare [49,50]. Furthermore, the integration of AI-assisted design and adaptive control strategies further optimizes energy conversion efficiency and operational stability, ensuring reliable performance under the stochastic energy conditions of the human body.

### Analysis of energy harvesting performance

**Figure 3.**
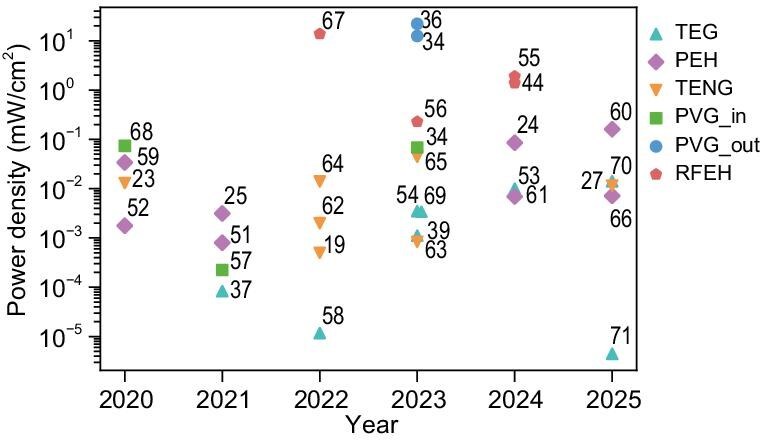
Power density of energy harvesters under human-like conditions [19,23–25,27,34,36,37,39,44,51–71].

The performance of different energy harvesters is dependent on the operating conditions. For example, the output power of PVG typically varies with light intensity, while the output power of TEG generally changes with the temperature gradient. These varying operating conditions make quantitative comparisons between different energy harvesters challenging. Therefore, studies conducted under human-like conditions were selected in this paper, and performance is analyzed within closely matched operating conditions (Fig. 3). Outdoor PVGs and RFEHs have power densities much higher than other generators. Indoor PVGs have a slightly higher power density than PEHs, which in turn is slightly higher than TENGs. However, power density alone is insufficient to determine their suitability for practical healthcare applications. In real-world scenarios, factors such as long-term stability, biocompatibility, and wearability are equally critical.

As summarized in Table 1, different energy harvesting approaches present distinct advantages and disadvantages when deployed on the human body. For instance, PVGs enable efficient light-to-electricity conversion but depend on illumination conditions. TEGs offer excellent long-term stability but are limited by relatively low power output and dependence on thermal gradients. PEHs provide high power density under dynamic motion but rely on continuous mechanical excitation. TENGs exhibit high voltage output and material versatility, yet may suffer from durability and environmental sensitivity. RFEHs support wireless and battery-free operation, although their efficiency and power output remain comparatively low.

**Table 1.** Summary of representative energy harvesters and their operating characteristics.

Ref.	Energy harvester	Reported performance	Long-term stability	Operating conditions	Biocompatibility
[70]	TEG	167 $\mathrm{\mu}$W Stable 0.4–0.8 V	Fully self-powered, continuous operation of a chronoamperometric glucose sensing module	Skin-ambient thermal gradients and a 1 mm tip height microneedle configuration for optimal dermal interfacing	Stretchable Ecoflex matrix *In vivo* rat model validation
[60]	PEH	3.10 mW/cm$^3$ 80.2 V and 9.04 $\mathrm{\mu}$A maximum	Self-powered, real-time biomechanical monitoring of dynamic human joint movements	Periodic mechanical impacts and varying joint bending deformations	Lead-free PVDF/CNF/ZnO electrospun nanocomposites Polyimide tape encapsulation for environmental stability
[27]	TENG	190 $\mathrm{\mu}$W peak power Max 166 V and 2.5 $\mathrm{\mu}$A at 5 Hz	Stable triboelectric output over 7 days and 1000 reciprocal slides, enabling machine learning-assisted human motion feature recognition	Low-frequency reciprocal sliding motion and limb bending	Electrospun PVA-Fe$_3$O$_4$ magnetic fiber membrane and nylon film Ecoflex encapsulation for wearable comfort
[34]	Indoor PVG	29.6% power conversion efficiency 140 $\mathrm{\mu}$W power output from a 2.0 cm$^2$ module	Steady power output over 24 h continuous operation and 2000 bending cycles, enabling continuous, battery-free multimodal physicochemical monitoring for over 12 h	Broad range of illuminance from dim indoor lighting to bright surgical rooms, alongside varying physical activities	Flexible quasi-2D perovskite structure with epoxy/PVC encapsulation Pb leakage strictly below drinking water limits, with high cell viability confirmed by *in vitro* tests
[36]	Outdoor PVG	22.10% power conversion efficiency 24.69 mA/cm$^2$ short-circuit current density and 1.15 V open-circuit voltage	T$_{80}$ lifetime of over 1570 h and 86% efficiency retention after 5000 bending cycles, enabling a wearable haptic device for pain sensation in virtual reality	Continuous AM 1.5 G solar illumination and ambient light with mechanical deformations including tension, compression, and bending	Aligned liquid crystal elastomer interlayer to preserve configurational integrity and prevent film fracturing Watch-like assembled device with microneedle-based arrays for safe human-machine interaction
[55]	RFEH	0.27% peak power transfer efficiency 6.76–7.04 dB improvement in transmission coupling strength	Stable electromagnetic coupling across various implant orientations based on rotation-insensitive and misalignment-resilient wireless power transfer	902-928 MHz industrial, scientific, and medical frequency band in radiative near-field region	0.02 mm biocompatible alumina coating encapsulation to prevent direct tissue contact Safe specific absorption rate strictly complying

Therefore, the selection of energy harvesting technologies should be guided by application-specific requirements rather than a single performance metric, further highlighting the necessity of system-level co-design in self-powered intelligence.

The material is important to the effectiveness of energy harvesting, as it defines the upper limit of the power output under ideal conditions. For example, the properties of piezoelectric material, for example, the piezoelectric coefficient, dielectric constant, and mechanical strength, directly affect the output power limit in PEH [22]. The output power of PEH is also influenced by factors such as mechanical stress and vibration frequency, particularly the magnitude and type of applied stress. The output power of TENG is determined by multiple factors, including material properties, contact area, applied pressure, and relative motion speed [72]. In daily human activities, the output power of PEHs and TENGs generally ranges from microwatts to milliwatts. Due to the low output power, high output impedance, and reliance on relative motion, the stability and power generation capacity are sometimes insufficient to directly power external devices.

The output of PVG is primarily influenced by material, size and lighting conditions. Under typical outdoor conditions (with sunlight intensity of 1000 $\mathrm{W/m^2}$, AM1.5 G [73]), solar cells can generate power ranging from several hundred milliwatts to several watts. In indoor lighting environments, such as LED or fluorescent lights (with typical illuminance around 1000 $\mathrm{lux }$[73]), solar cells usually converts several milliwatts to tens of milliwatts electrical power. While PVG is no longer limited to outdoor use, power density in indoor scenarios still needs to be improved. Notably, PVG is the energy harvesting method with a clear conversion efficiency metric, typically ranging from 10% to 22% for studied solar cells [73].

The output power of TEG is primarily influenced by the number of thermoelectric couples, the ZT value of thermoelectric materials and the temperature difference across the semiconductors. The ZT plays a decisive role in power generation performance, the latest reports have demonstrated flexible $\rm Ag_{2}Se$-based wearable thermoelectric films with a ZT value of 1.06 at 303 $\mathrm{K}$ room temperature [74]. However, most TEGs used in wearable/implantable applications for the human body only provide power in the range of a few microwatts to several milliwatts [37]. The generated electricity from TEG is normally accumulated in energy storage before supplying power to other elements. Nevertheless, due to a stable heat source served from the human body, and its robustness over mechanical wear, TEG is well-suited for long-term monitoring in medical applications [70].

The output power of RFEH is primarily influenced by signal strength, antenna design, and radio frequency, with signal strength often considered the important factor. Typical indoor Wi-Fi routers operate at a transmit power of 15–20 $\mathrm{dBm}$ ($\approx$ 30–100 $\mathrm{mW}$), while Bluetooth devices typically emit at 0 $\mathrm{dBm}$ to 4 $\mathrm{dBm}$ ($\approx$ 1–2.5 $\mathrm{mW}$). Considering free-space path loss, polarization mismatch, and impedance mismatch, the practically harvestable power at a distance of several meters is generally limited to the microwatt level. Milliwatt-level output can only be achieved under extremely short distances or under dedicated high-power radio frequency irradiation. Since signal transmitters are typically fixed devices, RFEHs offer relatively high stability, making it especially suitable for continuous monitoring and data transmission. However, RFEH for the human body is still in the early stages of exploration, and the impact of radiation on biological tissues (such as heating and safety concerns) limits its use [1].

### Harvesters on the human body

**Figure 4.**
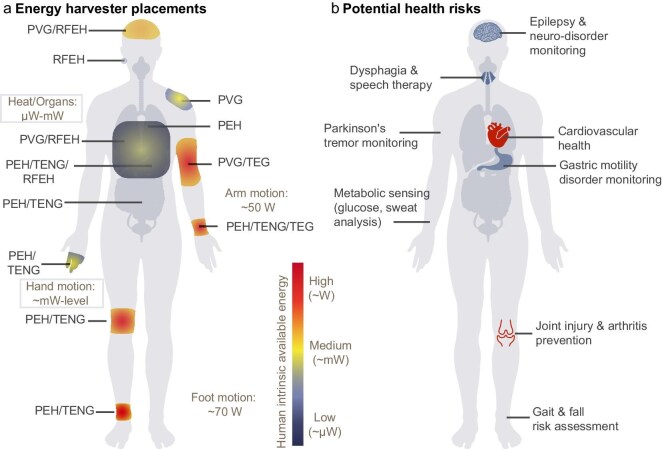
Strategic deployment of energy harvesters for personalized healthcare. (a) Map of human intrinsic available energy from different physiological sources and corresponding harvesting technologies. (b) Clinical application scenarios for self-powered intelligence at specific body locations.

In practical applications, energy harvesting, sensing, and intelligence must be co-optimized to meet specific healthcare needs. The type and power consumption of electronic components for different target applications affect the selection of energy harvesters, which in turn influences their installation location. More importantly, the efficiency of energy harvesting is strongly dependent on the anatomical location and corresponding physiological characteristics of the human body, as different regions provide distinct forms of accessible energy. Figure 4a summarizes the spatial distribution of human intrinsic energy and the corresponding placement of representative energy harvesters under realistic conditions. For areas such as the chest, head, shoulders, and arms, which are easily exposed to the light, PVGs are the most appropriate. Under sufficient light conditions, PVG remains stable with fewer limitations on system loads, making it suitable for a wide range of applications [34]. With the increasing demand for power supply to implantable devices, subcutaneous PVG has also been developed [68]. These subcutaneous PVGs are typically sensitive to specific wavelengths of light or require an active photon energy transmission system to be installed on the skin.

For joints such as the wrist, ankle, knee, and shoulder, which carry decent amount of kinetic energy, combining PEH and TENG is ideal. These harvesters can continuously collect various body movement energy, making them suitable for applications in athlete rehabilitation, sports injury recovery, and other healthcare applications [45]. In addition, some internal organs, such as the heart, stomach, and intestines, which have dynamic movement characteristics, are well-suited for PEH and TENG. These devices can be used to drive biochemical sensors for monitoring internal organ activities or power implantable devices, such as pacemakers [75,76].

The wrist and arm, with their relatively flat surfaces, experience significant temperature variations due to human activities (for example, cooling when rolling up sleeves or during wrist motion while running). These areas are well-suited for TEGs, as the large temperature differences can enhance the output performance. While TEGs are limited in terms of directly powering systems (for example, 2.5 $\mathrm{V}$ operating voltage for neurostimulators, medical devices in microwatts [69]), they can provide continuous power over time, allowing energy to be stored and redistributed.

The performance of RFEH is highly sensitive to the deployment location on the human body, as both the antenna orientation and the strong electromagnetic attenuation induced by biological tissues critically affect the effective received power. In implanted configurations, the high permittivity and conductivity of human tissues significantly deteriorate antenna radiation efficiency and impedance matching, leading to severe power degradation. As a result, most RFEHs are designed for epidermal deployment, such as behind the ear or on the chest, where the surface curvature is moderate and mechanical deformation is limited [77]. For brain–machine interface implants, although attenuation inevitably reduces the harvested power, such losses are generally considered acceptable in view of the substantial advantages over conventional wired power delivery. Traditional wired interconnects and rigid implants often induce considerable tissue damage and interfere with normal neural activity [15]. By eliminating direct physical interconnections, RFEH effectively alleviates long-term tissue injury caused by mechanical mismatch and micromotion.

These issues collectively highlight the importance of body-location-aware energy harvesting strategies, where the selection and optimization of harvesting mechanisms are tailored to specific anatomical regions and physiological conditions, enabling more efficient and reliable self-powered intelligence for healthcare.

Beyond energy availability, the selection of device location is also closely associated with specific clinical applications. As illustrated in Fig. 4b, different body locations enable distinct healthcare functions. For instance, energy harvesters integrated at the chest region can support cardiac and respiratory monitoring, which are essential for cardiovascular diseases and sleep-related disorders. Devices deployed on the wrist or upper limbs are well suited for activity tracking, tremor assessment, and rehabilitation monitoring, particularly for neurological conditions. In contrast, lower-limb locations, such as the knee and ankle, are highly relevant for gait analysis, mobility evaluation, and post-injury rehabilitation. In addition, energy harvesting approaches associated with internal organs may enable long-term monitoring of physiological signals for chronic disease management. While such location-specific deployment enables targeted healthcare functions, it also introduces additional design considerations related to the integration of energy harvesting units with the human body. Depending on the harvesting mechanism and placement, mechanical, electrical, or thermal interactions may arise, which can influence not only physiological signal acquisition but also the stability of sensing modules, the performance of electronic circuits, and overall wearing comfort.

Therefore, careful design of energy harvesting units, including flexible, and biocompatible configurations, as well as appropriate placement strategies, is required to minimize unintended coupling with other system components.

## FLEXIBLE AND SMART ELECTRONICS

Electronics are an important enabler of self-powered intelligence in personalized healthcare, bridging the gap between energy autonomy, physiological sensing, and intelligent data processing. Unlike conventional electronic systems, wearable and implantable healthcare devices must operate in close and prolonged contact with the human body, imposing stringent requirements on biocompatibility, mechanical compliance, and long-term reliability [78,79]. In addition, physiological signals often exhibit slow temporal dynamics and low bandwidth, creating opportunities for energy-efficient signal acquisition and processing. At the same time, the confined and delicate physiological environment places strict constraints on device size, weight, and power consumption, necessitating careful trade-offs between energy efficiency, computational capability, and system intelligence. Achieving stable operation under these coupled constraints represents a central challenge for self-powered intelligence. This section focuses on flexible and smart electronics, sensing and signal processing units, and non-Von Neumann architectures, highlighting how electronic system design is evolving to support long-term, low-power, and intelligent healthcare applications.

### Flexible electronics technology

For personalized healthcare, the materials used must be non-corrosive, and non-immunogenic to ensure long-term biocompatibility [78]. Additionally, flexibility (including bendable, stretchable properties) and reliability (encompassing operational stability and environmental durability) are essential, as devices must withstand physiological conditions while maintaining consistent performance. To ensure efficient operation within the constraints of low-power devices, avoiding excessively high data rates is essential. This allows the use of more versatile conducting tracks and interconnect solutions that may not be suitable for higher-speed electronics, thus balancing practical limitations with system performance requirements. When implementing flexible circuits, key considerations include material selection, circuit design, and the integration of flexible connections [78].

Material selection is a fundamental consideration in flexible design for ensuring biocompatibility. Connections that are loosely coupled with the body cannot provide meaningful information in personalized healthcare. Ultra-thin substrates facilitate accurate measurements and conformal contact with the skin [80,81]. Several polymer films have been used as flexible substrates for wearable/implantable devices, such as polycarbonate, thermoplastic polyurethane, polyethylene terephthalate, polydimethylsiloxane, polyethylene naphtholate, polyimide, and poly (3,4-ethylenedioxythiophene) polystyrene sulfonate. These materials possess excellent flexibility, thermal stability, and chemical resistance, enabling them to withstand repeated mechanical stresses. Polydimethylsiloxane, renowned for its outstanding biocompatibility, has been widely used in the biomedical field, and is one of the simplest and most popular candidates for flexible substrates [78]. Recently, hydrogels have gained attention due to their advantages in biocompatibility, mechanical properties, and self-healing capabilities [82,83]. For instance, a recent study reported an ionic hydrogel achieving an exceptional conductivity of 7.04 S/m, demonstrating superior performance in electrophysiological sensing compared to both conventional bio-hydrogels and commercial electrodes [84].

Beyond devices placed directly on the skin, integration into textiles and garments has emerged as a highly active research direction. A recent study demonstrated that two-dimensional microfabricated films can be converted into one-dimensional compliant electronic fibers using a spiral-neurostring [85]. This design enabled stable multi-channel single-neuron recordings in the mouse brain for up to four months. Textile-based electronic platforms enable mechanically robust, and unobtrusive sensing, providing an alternative route for comfortable long-term health monitoring without requiring continuous skin adhesion [86].

**Figure 5.**
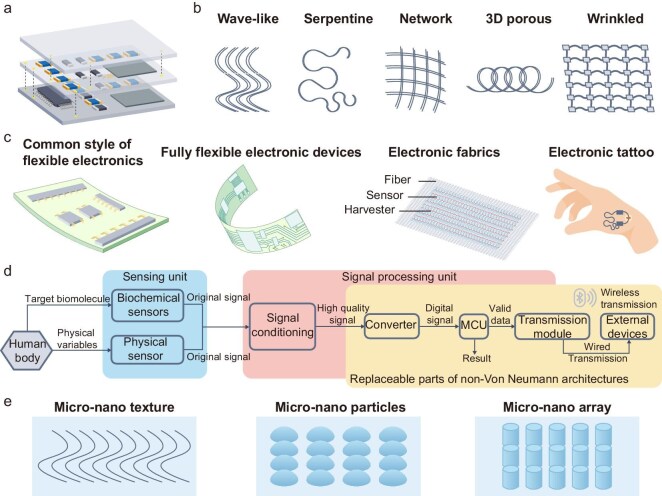
Flexible and smart electronics in self-powered intelligence for personalized healthcare. (a) Classical multilayer circuit design for miniaturization. (b) Connection structure that enhances flexibility. (c) Advanced flexible circuits. (d) Composition and function of the sensing and signal processing unit. (e) Typical micropatterns designed on the electrode surface.

Circuit layout design directly influences signal integrity and system stability, especially in dynamic environments. Electromagnetic interference must be carefully addressed, as it can degrade performance. One effective strategy for minimizing interference is to separate sensitive signal lines from power lines. Additionally, multi-layer routing technology can improve space utilization (Fig. 5a), making it ideal for integrating flexible substrates with miniature rigid electronic components to achieve complex high-performance integrated circuits [86,87].

Flexible connections, serving as the bridge for all system functions, need to be designed with consideration for their connection strength under bending, stretching, or other deformations. Polymer composites with excellent stretchability have become widely used as flexible interconnect materials [88]. These composite materials are typically made by doping polymer films with other conductive materials (for example, carbon nanotubes, Max phase, and graphene) to achieve functions like signal conversion and transmission. Carbon-based nanomaterials have attracted attention as doping materials due to their excellent electromechanical properties, chemical stability, and mature synthesis technologies [89]. However, encapsulation and doping often reduce the overall breathability and comfort of polymer films, so the final effect should be considered in the design. By designing the electrode shape, the geometric structure can be leveraged to enhance the flexibility of the circuit connections. Several geometric designs have been reported (Fig. 5b), including wave-like, serpentine structures, network patterns, 3D porous patterns, and wrinkled structures, which can achieve excellent electromechanical stability under strains of more than 100% [78]. These patterned electrodes, fabricated through simple manufacturing processes such as film deposition, etching, and photolithography, are conducive to large-scale production applications.

Various fabrication methods exist for flexible devices, such as screen printing [90], laser sintering [82], and chemical vapor deposition [91]. Most reported wearable/implantable devices are fabricated using a combination of rigid microelectronic components with flexible substrates and circuits (Fig. 5c). Existing studies have achieved fully flexible devices based on textile materials and functional tattoos, but their functionality remains relatively simple. With the development of flexible circuit elements such as printable thin-film transistors, flexible diodes, and flexible supercapacitors, future self-powered intelligence is expected to evolve toward fully flexible, biocompatible, and highly integrated intelligent systems.

### Sensing and signal processing units

Sensing and signal processing units are key components for efficient signal monitoring [1,3] (Fig. 5d). The sensor unit captures physiological signals from the body, while the signal processing unit transforms signals into reliable, user-understandable outputs. Given the slow variation of physiological signals, low-bandwidth signal collection and processing is both feasible and efficient, ensuring effective monitoring without the need for high sampling rates. The signal processing unit typically requires signal conditioning modules, analog-to-digital converters, microprocessor modules, and data transmission modules. To achieve effective physiological sensing and data processing with limited power, the circuits across different functional units must be co-designed in a holistic, system-level manner, a process that can be further facilitated by AI-assisted optimization and design strategies.

Human body sensors can be mainly categorized into physical sensors and biochemical sensors. Examples of the former include electromyographic sensors on the throat and accelerometers used for post-throat surgery rehabilitation monitoring [82], while the latter includes a sweat sensing system running on the fingertips which monitors metabolites such as lactic acid and levodopa [92], and aptamer-based nanosensors used for non-invasive female hormone monitoring [93]. To obtain more accurate sensing signals, micro-patterning on the surface of sensor electrodes is usually used (Fig. 5e). For instance, biomimetic temperature sensors designed based on the thermosensation ability of pit viper membranes can significantly improve temperature sensitivity [94], and pressure sensors inspired by the natural terrain of hills and valleys can detect pressure corresponding to the weight of a single grain of rice (16 $\mathrm{Pa}$) over the range of 0-2.6 $\mathrm{kPa }$ [95]. Micro-patterning design can significantly increase the surface area of the sensor electrode, thereby enhancing the electrical path between the sensitive layer and the underlying collector.

Improving the stretchability of sensors is also crucial for maintaining their stable functionality. Sensors used for personalized healthcare often need to closely conform to the skin or organ surfaces, and are prone to motion artifacts that interfere with signal accuracy during complex human movements. The ultra-thin structure of electrodes (with a total thickness of 10 $\mathrm{\mu m}$) can maintain close contact with the skin during movement, preventing relative motion or sliding. Studies have also incorporated small hole structures in the electrodes to improve adhesion and reduce peeling effects. For certain body joints, biomimetic designs combining layered and fiber structures can be used to achieve over 100% stretchability and meet the need for a close fit.

With the increasing demand for human body monitoring, multifunctional sensors are typically required. The most common approach is to tightly integrate multiple sensors together, but this leads to high-density and complex circuit connections, which compromises the system’s flexibility and reliability. Simultaneous exposure to various stimuli also introduce signal noise and crosstalk [87]. By simultaneously measuring the resistance and capacitance of ion conductors, signal decoupling can be achieved at the hardware level [96], which can be further enhanced by AI-assisted learning of signal features for efficient and adaptive decoupling. Technologies such as electronic fibers and microchips embedded within sensors can help reduce circuit wiring complexity and pre-process redundant data, thereby improving overall system efficiency. The sensor unit is usually connected to a signal processing unit for further data processing. Flexible thin-film transistors can construct key parts of signal processing units such as digital logic gates and amplifiers. A Wheatstone bridge circuit can be designed to detect small changes in resistance within the circuit and achieve temperature compensation within a certain range.

Data processing can mainly be divided into two modes: (i) cloud processing and (ii) on-chip processing. Cloud processing allows for the handling of large-scale data but requires a network connection, which introduces higher latency, increased power consumption, and the need for an active internet connection. On-chip processing, on the other hand, features low latency, high efficiency, and low power consumption, but is highly dependent on the computing capabilities of microchips, and has limited capability to employ large data sets. Considering the complexity and high-dimensional features of medical data, as well as the limitations in the computational power and power consumption of microchips, transmitting sensor data to the cloud for processing is currently a more common solution [82]. Low-power processors, such as the ARM Cortex-M series-based STM32, are suitable for deploying simpler algorithms for real-time signal processing. Some commercialized neuromorphic computing chips, such as Intel’s Loihi [2] and multiple versions of the SpiNNaker designed by the University of Manchester, can achieve more complex on-chip neural network computations [97]. Utilizing on-chip processing for data preprocessing and filtering, followed by cloud processing for more complex computations, long-term storage, and global analysis, will better leverage the advantages of both approaches.

Cloud processing involves wireless information transmission technologies, and significant improvements in transmission range, power consumption, and device size have made this technology widely applicable in personalized healthcare. Radio frequency identification devices based on near field communication (NFC) allow wireless information transmission over distances of up to 1 $\mathrm{m}$, complying with ISO/IEC 15693 standards. They can typically be used in conjunction with front-end systems. Currently, flexible transmission systems have been built using organic field-effect transistors that operate at 13.56 $\mathrm{MHz }$ [98]. Active wireless transmission systems, such as Bluetooth Low Energy (BLE) and Wi-Fi, can continuously establish connections with external devices or cloud servers. Most biological signal sampling frequencies are below 1 $\mathrm{kHz}$, and NFC or BLE-based transmission systems can already meet the requirements [92]. When high-frequency data transmission is needed, Wi-Fi transmission is required. However, this approach often causes information bottlenecks, reducing the real-time processing capability of self-powered intelligence.

### Non-Von Neumann architecture

**Figure 6.**
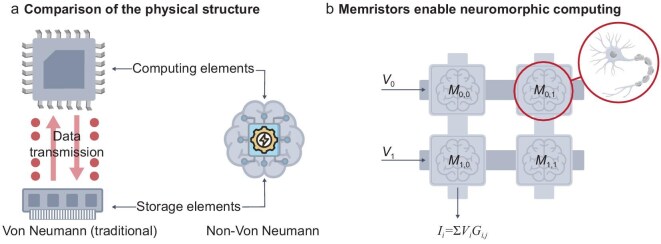
Non-Von Neumann architecture and typical applications. (a) Architecture comparison between Von Neumann and non-Von Neumann computing. (b) In-memory computing using memristor crossbar arrays. $G_{i,j}$ is the conductivity of the memristor in the *i*th row and *j*th column; $M_{0,0}$ is the memristor at the 0th row and 0th column.

Considering the vast amount of medical data and the real-time requirements of personalized healthcare, both cloud processing and on-chip processing modes face ‘computational bottlenecks’. This limitation is due to the traditional Von Neumann architecture design (which is used in most devices such as CPUs and GPUs), where the computing elements and storage elements are physically separated (Fig. 6) [99]. Researchers have attempted to overcome this bottleneck by designing more compact computing architectures named non-Von Neumann architectures. The key feature of non-Von Neumann architectures is the integration of storage and computation units within the same element [100,101]. Memristors are among the most well-known components with a non-Von Neumann structure and are currently the most suitable elements for achieving built-in memory volatility and plasticity [100]. A memristor is a device that can change and remember its resistance state, which can store and modify data while processing it, and achieves the characteristic of integrated storage and computation.

Non-Von Neumann architectures reduce data transmission latency and energy consumption while improving processing efficiency through data parallelism [100,102]. This offers a powerful solution to the low-power and miniaturization challenges faced by self-powered intelligence. Recent work has introduced a memristor-based compressed-sensing accelerator that achieves 11.22 times speedup and 30.46 times energy savings compared with state-of-the-art CMOS hardware [103]. Currently, ‘neuromorphic computing’ based on non-Von Neumann architectures has been validated and can be used to complete a variety of complex AI tasks [104]. Neuromorphic computing works by simulating biological neurons using circuits similar to the human neural structure, and has already been applied to real-time monitoring and analysis of electrocardiograms (ECG) [105] and cancer diagnosis systems [97]. However, the design and implementation of non-Von Neumann architectures are highly complex and require specialized hardware and software support. Non-Von Neumann devices, like memristors, have not yet reached the same level of technological maturity as transistors. Specific non-Von Neumann structures must be designed for specific algorithms, meaning their versatility is still far behind that of traditional structures.

The high energy efficiency, real-time processing capability, low power consumption, and small size of non-Von Neumann architectures provide promising solutions for self-powered intelligence in healthcare. Although development in this field is still in its early stages and faces multiple challenges, such as design complexity and architecture versatility, non-Von Neumann architectures will be indispensable for the future of self-powered intelligence in personalized healthcare. Thus, this area is worth further exploration and development.

## AI FOR PERSONALIZED HEALTHCARE

**Figure 7.**
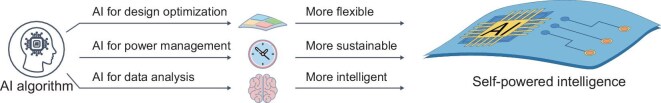
AI algorithms for self-powered intelligence.

AI plays a key role in transforming self-powered devices from passive data collectors into proactive healthcare systems. Under strict constraints of limited energy supply and compact hardware, lightweight and on-device AI enables real-time interpretation of multimodal physiological signals, early anomaly detection, and personalized health assessment, which are critical for continuous healthcare and society-level health management. In the context of self-powered intelligence, AI must be co-designed with energy harvesting and electronic systems, rather than treated as an independent software layer. Algorithmic efficiency, adaptive power management, and robustness under fluctuating energy availability become central considerations for practical deployment. This section focuses on the design optimization of AI-assisted structures, intelligent power management for self-powered operation, data processing and analysis for healthcare, and lightweight deployment of algorithms (Fig. 7).

### Intelligent design optimization

**Figure 8.**
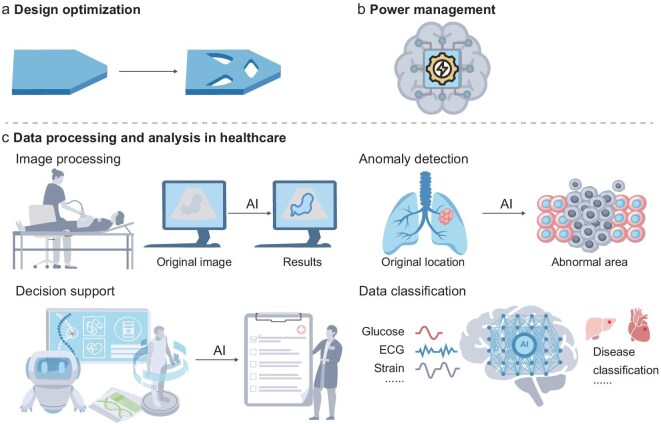
AI algorithms for self-powered intelligence in personal healthcare. (a) AI can assist designers in the efficient design of materials and devices, offering optimization solutions. (b) AI can improve power management efficiency in the miniaturization of wearable/implantable devices. (c) AI algorithms are smart tools in medical data processing and analysis, enhancing diagnostic efficiency and supporting decision-making for doctors.

Self-powered intelligence operating in the human environment imposes stringent constraints on size, weight, and power, while inherently requiring integrated AI capabilities to realize intelligent sensing and autonomous decision-making. When designing such devices based on researchers’ experience and experimental trial-and-error methods, a significant amount of time and effort is needed to adjust the device structure, material selection, and manufacturing schemes. These challenges can be formulated as multi-objective optimization problems subject to boundary conditions. In this context, AI provides an effective means to address such complex optimization tasks (Fig. 8a).

In structural design, AI algorithms can simulate and analyse various topological structures based on design objectives, utilizing algorithms such as graph neural networks and Bayesian optimization to rapidly identify the optimal topology. The topologies designed by AI algorithms can even outperform those designed by human experts in terms of quality and performance [106]. For example, Random Forest (RForest) has been used to learn and predict the efficiency of thin-film solar cells with different absorber layer thicknesses, achieving an optimized absorber layer thickness of 350 $\mathrm{nm}$, and experimentally verifying a maximum power conversion efficiency of 17.42% [107]. Physics-informed surrogate neural network (PINN) has been used for optimizing diode-based interfaces in pacemaker energy harvesting, an average error of 0.03 $\mathrm{V}$ between prediction and measurement can be achieved [108]. Intelligent material selection is also an essential part of the system design process. By leveraging machine learning and data mining techniques, researchers can quickly identify suitable materials from extensive material databases for specific applications. For example, using parameters such as electronegativity, ionic radius, ideal bond length, and bandgap energy levels, intelligent algorithms like density functional theory, RForest, and Support Vector Machine (SVM) can be employed to select efficient energy-harvesting materials from a wide range of candidates. This AI-based material selection strategy significantly shortens development cycles and enhances research efficiency. In addition to directly selecting materials, researchers can also use AI to simulate the operational environment, providing better prediction of material performance [109]. During system design, parameter optimization is another crucial issue. Given an initial structure or material category, AI can continuously adjust and optimize structural or material composition parameters based on real-world application data, improving the overall design [110]. For instance, a combination of neural networks and genetic algorithms can be used to solve the optimal design parameters for PEHs [110]. AI can also sense and adjust the process environment, or predict the impact of different manufacturing processes on material performance [111], helping researchers determine the optimal manufacturing process.

### Power management for self-powered device

Compared to power management in large power grids, self-powered intelligence operates at much lower energy levels, typically ranging from milliwatts to watts, posing unique challenges in small-scale power management. These challenges include real-time status monitoring, usage frequency, and energy harvesting performance. Voltage management becomes important for power management in low-power environments, as devices often operate at varying voltage levels. The conversion between different voltage, due to the diversity of energy harvesters, applications, and storage devices, introduces additional complexity and losses. Traditional power management technologies, designed for large-scale systems with complex network, cannot be directly applied to such low-power systems due to their lack of sensitivity to voltage fluctuations, power loss and real-time requirements. Since self-powered intelligence for personalized healthcare is still in its early stages, most studies have focused on biological signal sensing, flexible materials, and multi-energy harvesting technologies, with limited attention on power management. As self-powered intelligence is deployed in real-world settings, the inherent tension between limited energy availability and long-term operation shifts system design toward efficient voltage and power control. In this context, small-scale human power networks require tight coordination among load prediction, power scheduling, and power management, which can be jointly enabled by AI algorithms that learn usage patterns, anticipate energy demand, and dynamically optimize power allocation under time-varying conditions.

Load prediction can estimate the power demand at different time periods by analyzing user behavior patterns (such as activity levels, rest, etc.), which is suitable for improving system efficiency in energy-constrained environments [112]. CNN-based network models and variations of SVM are used to predict short-term power demand for devices [113].

Power intelligent scheduling determines which tasks or loads should be prioritized for power supply at specific time points to meet immediate demands, while optimizing power usage efficiency and extending device operation time. This requires careful power management to ensure that power is supplied within the correct voltage ranges for each task. Algorithms such as the knowledge sharing algorithm allow researchers to manage power across complex human power networks and optimize device power consumption [114]. This approach is particularly suitable for self-powered systems, where maintaining stable voltage levels is essential for reliable operation.

Intelligent power management enables real-time monitoring of system power, formulation of control strategies, and management of various power sources to ensure power supply under different operating conditions [115]. For intelligent power management, load prediction provides the data support, while power intelligent scheduling converts these predictions into specific action strategies. Additionally, intelligent power management is responsible for the energy conversion efficiency in the circuit, such as determining whether maximum power point tracking (MPPT) is needed and controlling the related MPPT algorithms through the controller [116].

As the demand for prolonged and stable energy supply increases, multi-source energy harvesting has emerged as a promising solution. Yet, most existing designs emphasize hardware-level integration of heterogeneous power inputs via rectifier circuits and power converters, with limited intelligence in coordinating multiple sources. This lack of AI-driven management hinders optimal power integration and distribution, particularly in maintaining stable and device-compatible voltage outputs under time-varying and asynchronous energy inputs.

### Data processing and analysis for healthcare

AI algorithms are powerful tools for data processing and analysis (Fig. 8c). Integrating AI into earlier stages of the sensing pipeline can enable adaptive sampling, noise suppression, and signal enhancement, thereby improving data quality under resource-constrained conditions. More importantly, such integration facilitates closed-loop sensing paradigms, in which real-time feedback dynamically adjusts sensing configurations and acquisition strategies according to signal quality and task requirements, enhancing both data fidelity and energy efficiency.

In personalized healthcare, data are derived from heterogeneous sources, including physiological, physical, and biochemical signals acquired by wearable and implantable sensors, as well as medical images obtained from clinical imaging systems. These multimodal data necessitate tailored AI models for effective representation, classification, and interpretation. To support the development and validation of such algorithms, large-scale and standardized datasets have been established through international initiatives, such as the Cancer Imaging Archive [117] and the Cardiac Atlas Project [118], providing critical resources for advancing AI-driven medical applications.

Recent advances in AI-enabled sensing systems further highlight the integration of intelligent preprocessing and adaptive acquisition within unified frameworks, marking a transition from passive data collection toward active, context-aware, and self-optimized sensing strategies [119].

In classification tasks, algorithms such as SVM and RForest are classical and effective. Through continuous monitoring of ECG, respiration, blood volume pulse, skin conductivity, and skin temperature [120], algorithms such as K-Nearest Neighbour (KNN) and RForest can be used to classify sensor data and distinguish between different emotional stress states, greatly assisting in personal mental health monitoring [121]. Decision Trees (DT) can also be used to train data obtained from wearable devices to classify interstitial glucose levels [122].

In anomaly detection tasks, algorithms are required to identify rare events within high-dimensional data, which remains a fundamental challenge in medical applications. Abnormal samples often correspond to distinct disease characteristics, yet their availability is typically limited. Under such extremely imbalanced data distributions, deep neural networks tend to overfit or memorize dominant patterns, leading to degraded performance when encountering previously unseen cases [3].

To address these challenges, a variety of machine learning approaches have been explored. Traditional algorithms such as SVM and random forests have been applied to handle rare-event detection, for example by monitoring human movement data to automatically identify abnormal states and provide timely alerts to healthcare providers [123]. More recently, deep learning frameworks combined with transfer learning have demonstrated improved generalization and data efficiency. For instance, adaptive long short-term memory (LSTM) networks integrated with transfer learning have been used for early warning of epileptic seizures through electroencephalography signal analysis [124]. In addition, efficient learning paradigms such as broad learning systems have been proposed for wearable sensing applications, including cuffless blood pressure estimation, achieving competitive accuracy with significantly reduced training time [125].

For decision support tasks, reinforcement learning (RL) has increasingly demonstrated its potential, particularly in real-time monitoring and intervention. RL can recommend optimal treatment plans based on patient data. It works by learning the best actions through interactions with the environment, which correspond to patient responses, disease progression, and medical interventions taken by doctors [126]. By integrating the expertise of human doctors, RL can even generate safe and reliable treatment strategies for conditions such as sepsis, potentially rivalling those developed by clinical experts [127].

The diverse nature of tasks in personalized healthcare have been summarized in Table 2, categorized by task characteristics and the algorithms employed.

**Table 2.** Suitable AI algorithms for different task types.

Task types	Task examples	Representative AI methods	Algorithm features
Data classification	Disease diagnosis, patient stratification	RForest, SVM, KNN, DT [121,122]	Powerful classification capabilities, suitable for multi-category classification tasks
Anomaly detection	Detect lesions in medical images and monitor abnormal physiological parameters	RForest, SVM [123]	Has the ability to learn with small samples and suitable for discovering rare events
		LSTM, RNN [124]	Suitable for analysing time series data
Decision support	Diagnostic suggestions and treatment recommendations	RL, DT [126,127]	Can handle complex decision-making problems and suitable for decision optimization in dynamic environments
Predictive analytics	Disease prediction and patient recovery prediction	Statistical regression methods, CNN + RNN hybrid architecture [128,129]	Suitable for continuous data and can capture long-term dependencies
Image processing	Medical image segmentation, image enhancement	CNN, ResNet, GAN [130,131]	Optimize image quality and feature extraction, and be able to generate new image data
Text processing	Medical literature analysis, medical record text mining	BERT, LSTM, GPT [132]	Has strong ability to process text data and suitable for extracting information from unstructured data

Beyond the different task types mentioned above, inter-individual variability in physiological signals introduces an additional layer of complexity, arising from differences in anatomy, lifestyle, disease progression, and sensor placement. To improve robustness and generalization across subjects, AI models increasingly incorporate personalization-aware strategies. Techniques such as domain adaptation and meta-learning enable rapid calibration to unseen individuals with limited data, while lightweight subject-specific fine-tuning modules allow efficient deployment under resource constraints. Furthermore, representation learning approaches, including feature disentanglement, help separate invariant physiological patterns from individual-specific variations, thereby enhancing cross-subject consistency.

These algorithmic advances can be further integrated with adaptive sensing frameworks, where model feedback dynamically guides data acquisition and preprocessing in a subject-aware manner. Such closed-loop optimization enables the joint improvement of sensing quality and model performance, supporting more reliable and energy-efficient personalized healthcare systems.

Large model technologies possess stronger feature extraction and data processing capabilities [133]. Fine-tuning large and small models, as well as training domain-specific large models, allows the heterogeneity and high dimensionality of healthcare data to be fully leveraged. Some studies have already used GPT-3.5 to build bioelectronic interfaces, recognizing both real and simulated speech to identify pronunciation patterns for people with hearing and speech impairments[134]. However, large models also come with high demands on computational resources, data privacy, and algorithmic ethics problems. Deploying large models in self-powered intelligence is highly challenging. The complexity of large models reduces AI interpretability, affecting both patients’ and healthcare professionals’ understanding of model results. Issues such as decision fairness in model training, responsibility in model usage, and legal gaps further increase the challenges of applying large models in self-powered intelligence.

### Lightweight deployment of algorithms

**Figure 9.**
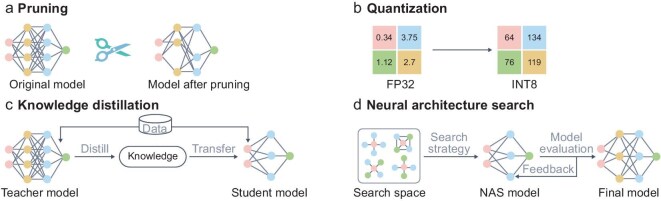
Common methods for lightweight deployment, with most improvements based on these four fundamental techniques. (a) Removal of redundant parameters and connections through pruning. (b) Reduced numerical precision for efficient inference via quantization. (c) Knowledge transfer from teacher to student models through distillation. (d) Automated optimization of network architectures using neural architecture search.

Self-powered intelligence imposes stringent constraints on power consumption and computational resources, particularly for local, on-device deployment, where continuous operation must be sustained under severely limited energy budgets [135]. An inherent trade-off arises among model complexity, inference latency, and power consumption: more complex models generally provide higher accuracy but increase computational load, latency, and energy use, which are critical considerations for energy-constrained systems. In such scenarios, AI algorithms are implemented in a lightweight manner, with reduced model complexity and computational demand, to ensure efficient and reliable performance in resource-constrained environments. A range of lightweighting strategies have been developed, with common methods, including model pruning [136], knowledge distillation [137], Neural Architecture Search [138], and quantization [139] (Fig. 9). The first three methods aim to reduce memory usage by exploiting sparsity in network connections, while quantization further reduces memory by appropriately lowering bit precision [97].

Single-layer LSTMs with shortened input sequences have been shown to reduce spatiotemporal computational demands, enabling deployment on microcontroller-based computing modules. The model can estimate metabolic rate from human joint motion data, maintaining an average absolute percentage error of 9%–10% [16]. Compression learning combining compressed sensing matrices with 1D CNNs significantly reduces network parameters, allowing deployment on microcontrollers such as MSP432 for epilepsy seizure detection, achieving an accuracy of 96.44% at a compression ratio of 0.1 [140].

The staged CNN is another method to reduce power consumption. In the first stage, a simple neural network performs a rough binary classification task, while only in the presence of abnormal signals does a more complex network get activated. For example, a two-stage CNN achieved a low-power arrhythmia classifier, reaching 98.6% accuracy in arrhythmia detection across 1320 ECG samples [141].

Beyond enabling efficient on-device inference for physiological data analysis, lightweight AI algorithms also make it feasible to perform *in situ* monitoring of sensor reliability and system health under strict energy constraints. Learning-based models can identify abnormal signal patterns caused by sensor drift, aging, fouling, or unstable skin contact, distinguishing them from genuine physiological variations. Such on-device diagnostic capability is particularly important for self-powered wearable and implantable systems, where long-term autonomous operation limits opportunities for recalibration or maintenance.

Hardware acceleration can further alleviate the trade-offs among model complexity, latency, and power consumption through coordinated circuit-algorithm design. Neuromorphic devices are typical adaptations, allowing the available resources to remain minimal while improving energy efficiency [97]. Easier-to-implement hardware designs can start from the data storage optimizations, such as using 16-bit floating-point representations in the hardware network, accelerating convolution layers, pooling layers, and fully connected layers, enabling lightweight CNN for arrhythmia detection in wearable devices, achieving a classification accuracy of 97.69% [142].

Building upon these advances, recent studies have demonstrated the feasibility of integrating self-powered energy harvesting, flexible electronics, and artificial intelligence into unified healthcare systems. For instance, self-powered wearable devices have demonstrated continuous and battery-free physiological monitoring by harvesting energy from human motion [143], heat, or biochemical processes [144]. In parallel, AI-enabled wearable technologies have shown strong potential in clinical applications, enabling real-time analysis of physiological signals [119,145] and supporting patient monitoring, risk prediction, and clinical decision-making [146]. When integrated with low-power hardware and lightweight models, these systems can perform tasks such as activity recognition, anomaly detection, and personalized health assessment under constrained energy conditions. These examples demonstrate that self-powered intelligence is transitioning from isolated functional modules toward integrated, application-oriented systems, where energy harvesting, sensing, and intelligence are co-designed for sustainable and personalized healthcare.

Although lightweight techniques enhance the applicability of models on resource-limited devices, practical deployment still faces many challenges, such as performance degradation, reduced interpretability and hardware constraints.

The lightweighting process may reduce model performance, particularly for complex medical signals, and can diminish interpretability, affecting clinical trust. As large model technologies advance, their storage and energy demands increasingly conflict with the constraints of self-powered systems. Overall, these challenges highlight the critical need to balance model complexity, inference latency, and power consumption through lightweighting and hardware-aware strategies to enable practical AI deployment in personalized healthcare.

## CHALLENGES AND FUTURE DIRECTIONS

**Figure 10.**
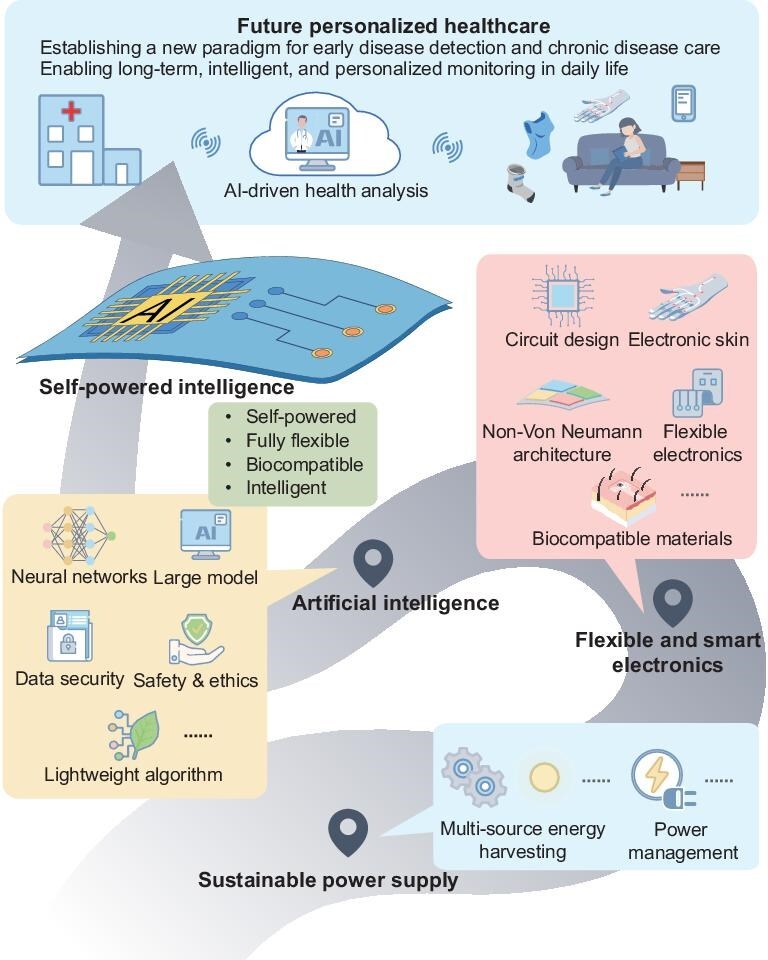
Potential challenges for the future development of self-powered intelligence.

Achieving long-term, autonomous self-powered intelligence remains a central challenge in personalized healthcare, particularly when integrating hybrid energy harvesting, biointegrated electronic systems, and lightweight intelligent algorithms into a unified and reliable platform. While recent advances have significantly expanded the capabilities of wearable and implantable devices for physiological monitoring, the transition of self-powered intelligence from laboratory demonstrations to practical clinical applications remains limited by system-level constraints, long-term reliability, and real-world operating conditions (Fig. 10).

### Sustainable power supply

Energy harvesting technologies provide promising solutions for self-sustainable power supplies, gradually reducing dependence on conventional batteries in wearable and implantable healthcare systems. Beyond extending device longevity, their clinical value lies in enabling permanent, maintenance-free operation. Unlike traditional battery-powered implants, which require invasive secondary surgeries for replacement once exhausted, self-powered systems can operate continuously without intervention, thereby reducing patient trauma and long-term healthcare costs. By eliminating bulky, rigid, and potentially toxic batteries, self-powered intelligence further mitigates risks such as chemical leakage and thermal runaway, while allowing more conformal and biocompatible device designs that enhance patient compliance. Nevertheless, achieving stable and continuous operation requires more than improvements in energy conversion materials and flexible circuit design. Intelligent energy management at the system level is increasingly important to accommodate the dynamic, heterogeneous, and often unpredictable conditions of the human body. In addition to constraints imposed by size and weight, maintaining stable power delivery in the presence of variable physiological activities and environmental fluctuations poses significant challenges.

Hybrid energy harvesters further expand the potential for long-term and reliable operation by exploiting complementary energy sources. Conceptual device designs illustrate how multiple energy conversion mechanisms can be integrated within a single platform, such as combining piezoelectric, triboelectric, and photovoltaic elements in a flexible patch or implant. Intelligent energy management modules and adaptive control architectures enable real-time allocation of harvested energy to meet AI computational requirements, ensuring stable and efficient operation. Careful design is needed to match the energy harvesting capacity with the device’s computational load, particularly for AI-driven tasks. Yet, most existing studies emphasize independent control circuits for individual inputs, without fully utilizing their inherent complementarities. Future progress will likely depend on AI-enabled strategies for multi-source energy prediction, adaptive control, and coordinated coupling, enabling more efficient and robust power integration tailored to long-term healthcare monitoring.

### Flexible and smart electronics

Flexible electronics technology enables wearable/implantable capabilities in self-powered intelligence, encompassing energy harvesters, sensing interfaces, and signal processing circuits tailored for human-centered applications. Despite rapid progress, challenges remain in material selection, as limited durability, poor thermal stability, and high viscosity often hinder the fabrication of devices with reliable and reproducible characteristics. Beyond materials and mechanics, system-level power consumption emerges as a key constraint, particularly for long-term autonomous operation under the stringent energy budgets of personalized healthcare. AI-enabled system architectures can play a pivotal role by adaptively optimizing sensing, computation, and communication strategies according to physiological conditions and clinical requirements. Wirelessly interrogated approaches, such as RFID-based systems, further reduce communication-related power consumption by eliminating the need for active transmitters. When combined with AI-driven decision-making, provide a viable pathway toward personalized, long-term health monitoring with minimal energy overhead.

Clinical translation further complicates system design due to anatomical specificity. Devices targeting the skin, brain, heart, or peripheral nerves require distinct mechanical properties and must carefully address biocompatibility and safety considerations. Emerging concepts such as transient electronic devices offer promising solutions by reducing the need for secondary surgical removal, though their long-term stability and functional predictability require further investigation.

Non–von Neumann architectures offer unique advantages for self-powered intelligence in personalized healthcare, including parallel processing, in-memory computation, and event-driven operation. These features are well aligned with continuous physiological monitoring and personalized data analysis under energy constraints. By enabling efficient, in-memory, and event-driven computation, such architectures are well aligned with the requirements of continuous physiological monitoring and individualized data analysis. However, practical implementation remains at an exploratory stage, facing challenges in material compatibility, thermal stability, energy management, and integration with existing biomedical systems. The lack of standardized design frameworks and regulatory guidelines further limits clinical transition and large-scale deployment.

### AI under ultra-low-power constraints

AI constitutes a core functional component of self-powered intelligence, enabling data interpretation, decision assistance, and autonomous operation under strict energy constraints. Continuous advances of AI, particularly in data analysis and decision assistance, have provided opportunities for the development of self-powered intelligence. Studies in lightweight algorithms have enabled complex AI models to run on devices with extremely low power consumption. Beyond data analysis, lightweight on-device AI can further support continuous self-diagnosis of sensor and system integrity, that is an often overlooked yet critical requirement for the long-term autonomy of self-powered intelligence applying for healthcare.

At the same time, the practical deployment of AI in self-powered intelligence introduces several challenges, particularly in the personalized healthcare. Security, trustworthiness, and regulatory compliance remain central concerns, as continuous physiological monitoring involves the collection, transmission, and processing of highly sensitive personal data. Ensuring data privacy, secure communication, algorithm transparency, and fairness is therefore essential for building reliable and clinically acceptable systems. These requirements are especially stringent in medical scenarios, where compliance with healthcare regulations and standards is necessary to guarantee patient confidentiality and trust. Future efforts should emphasize privacy-preserving strategies, such as on-device intelligence, federated learning, and secure data transmission protocols, to enable safe and reliable operation.

In parallel, the rapid evolution of large-scale AI models poses additional challenges for self-powered intelligence. State-of-the-art models typically require substantial computational resources, often relying on thousands of GPUs for training and inference, which is incompatible with the limited energy and hardware budgets of self-powered systems. The increasing model complexity and parameter scale demand more advanced lightweight strategies to balance performance with strict constraints on power consumption, latency, and hardware footprint. In personalized healthcare applications, a key challenge lies in effectively leveraging the generalization capability of large models while ensuring high accuracy, real-time responsiveness, and ultra-low power operation.

### Clinical translation considerations

Despite the promising progress in laboratory prototypes, the transition of self-powered intelligence into clinical practice is hindered by significant barriers in manufacturing scalability and system-level integration. Current fabrication methods often rely on small-scale processes that suffer from high unit costs and limited reproducibility. To achieve widespread clinical adoption, a shift toward high-throughput batch production is essential to ensure the consistency and economic viability required for population-level healthcare. Furthermore, the integration complexity inherent in merging heterogeneous units, including energy harvesters, flexible sensors, and low-power processing circuits, demands robust strategies for maintaining signal integrity and efficient data coupling within the dynamic and unpredictable biological environment.

Beyond fabrication and architectural constraints, the long-term reliability and service life of these systems remain critical concerns for chronic clinical applications. While temporary monitoring can be achieved with relatively simple designs, permanent or long-term implants require sophisticated encapsulation techniques to withstand the corrosive biochemical environment of the human body. Ensuring sustained biocompatibility while preventing biofouling or material degradation is vital to maintaining the high-fidelity performance of sensing and power delivery over extended periods. Addressing these multifaceted challenges in reliability and maintenance-free operation is a prerequisite for self-powered intelligence to move beyond a proof-of-concept stage toward reliable, long-term personalized healthcare solutions.

## CONCLUSIONS

Self-powered intelligence represents a transformative paradigm for personalized healthcare, enabling continuous sensing, intelligent analysis, and autonomous operation without reliance on conventional batteries, representing a fundamental shift from episodic monitoring to continuous and adaptive health management. By integrating energy harvesting technologies, flexible and biointegrated electronics, and lightweight AI algorithms, significant progress has been made toward wearable and implantable systems capable of long-term healthcare monitoring and decision support. This review systematically summarizes recent advances across these interconnected domains and highlights their collective role in shaping next-generation healthcare technologies.

From a physical perspective, future implementations may evolve toward chipless, battery-free, and mechanically flexible forms, further enhancing wearability and user comfort. Despite these advances, a fundamental limitation remains: the energy density that can be harvested from the human body and surrounding environment is inherently low, often insufficient to continuously power healthcare devices. To address this constraint, hybrid energy harvesting approaches that combine complementary sources, along with intelligent energy management, adaptive computation, ultra-low-power electronics, and lightweight AI algorithms, are considered the promising pathways. Event-driven or intermittent operation strategies can further optimize energy usage, enabling meaningful functionality even under stringent energy budgets. Future progress is therefore expected to rely on the synergistic integration of energy harvesting, circuit design, and intelligent algorithms, rather than relying on a single technology breakthrough. Beyond technological innovation, ethical considerations, safety, and regulatory frameworks will play an important role. Realizing the full potential of self-powered intelligence may require continued collaboration among AI researchers, materials scientists, device engineers, clinicians, and regulatory bodies, transiting laboratory breakthroughs into scalable, reliable, and accessible healthcare solutions, ultimately advancing continuous personalized healthcare and society-level health management.

## References

[1] Zhao C, Park J, Root SE et al. Skin-inspired soft bioelectronic materials, devices and systems. Nat Rev Bioeng 2024; 2: 671–90. 10.1038/s44222-024-00194-1

[2] Brasier N, Wang J, Gao W et al. Applied body-fluid analysis by wearable devices. Nature 2024; 636: 57–68. 10.1038/s41586-024-08249-4 39633192 PMC12007731

[3] Ates HC, Nguyen PQ, Gonzalez-Macia L et al. End-to-end design of wearable sensors. Nat Rev Mater 2022; 7: 887–907. 10.1038/s41578-022-00460-x 35910814 PMC9306444

[4] Mishra T, Wang M, Metwally AA et al. Pre-symptomatic detection of covid-19 from smartwatch data. Nat Biomed Eng 2020; 4: 1208–20. 10.1038/s41551-020-00640-6 33208926 PMC9020268

[5] Mahato K, Saha T, Ding S et al. Hybrid multimodal wearable sensors for comprehensive health monitoring. Nat Electron 2024; 7: 735–50. 10.1038/s41928-024-01247-4

[6] Schroeder TBH, Guha A, Lamoureux A et al. An electric-eel-inspired soft power source from stacked hydrogels. Nature 2017; 552: 214–8. 10.1038/nature24670 29239354 PMC6436395

[7] Li Y, Chen Z, Liu Y et al. Ultra-low frequency magnetic energy focusing for highly effective wireless powering of deep-tissue implantable electronic devices. Natl Sci Rev 2024; 11: nwae062. 10.1093/nsr/nwae062 38628571 PMC11020258

[8] Nguyen PQ, Soenksen LR, Donghia NM et al. Wearable materials with embedded synthetic biology sensors for biomolecule detection. Nat Biotechnol 2021; 39: 1366–74. 10.1038/s41587-021-00950-3 34183860

[9] Tringides CM, Vachicouras N, de Lázaro I et al. Viscoelastic surface electrode arrays to interface with viscoelastic tissues. Nat Nanotechnol 2021; 16: 1019–29. 10.1038/s41565-021-00926-z 34140673 PMC9233755

[10] Gupta AS, Patel S, Premasiri A et al. At-home wearables and machine learning sensitively capture disease progression in amyotrophic lateral sclerosis. Nat Commun 2023; 14: 5080. 10.1038/s41467-023-40917-3 37604821 PMC10442344

[11] Yang Z, Mitra A, Liu W et al. Transformehr: transformer-based encoder-decoder generative model to enhance prediction of disease outcomes using electronic health records. Nat Commun 2023; 14: 7857. 10.1038/s41467-023-43715-z 38030638 PMC10687211

[12] Pyun KR, Kwon K, Yoo MJ et al. Machine-learned wearable sensors for real-time hand-motion recognition: toward practical applications. Natl Sci Rev 2023; 11: nwad298. 10.1093/nsr/nwad298 38213520 PMC10776364

[13] Gao W, Emaminejad S, Nyein HYY et al. Fully integrated wearable sensor arrays for multiplexed in situ perspiration analysis. Nature 2016; 529: 509–14. 10.1038/nature16521 26819044 PMC4996079

[14] Lu L, Wu J, Zhang Y et al. Noncontact 3d gesture recognition enabled vr human-machine interface via electret-nanofiber-based triboelectric sensor. Nano Res 2025; 18: 94907924. 10.26599/NR.2025.94907924

[15] Das R, Moradi F, Heidari H. Biointegrated and wirelessly powered implantable brain devices: a review. IEEE Trans Biomed Circuits Syst 2020; 14: 343–58. 10.1109/TBCAS.2020.2966920 31944987

[16] Yuan J, Zhang Y, Wei C et al. A fully self-powered wearable leg movement sensing system for human health monitoring. Adv Sci 2023; 10: 2303114. 10.1002/advs.202303114 PMC10582417 37590377

[17] Aubin CA, Gorissen B, Milana E et al. Towards enduring autonomous robots via embodied energy. Nature 2022; 602: 393–402. 10.1038/s41586-021-04138-2 35173338

[18] Gao M, Wang P, Jiang L et al. Power generation for wearable systems. Energy Environ Sci 2021; 14: 2114–57. 10.1039/D0EE03911J

[19] Graham SA, Patnam H, Manchi P et al. Biocompatible electrospun fibers-based triboelectric nanogenerators for energy harvesting and healthcare monitoring. Nano Energy 2022; 100: 107455. 10.1016/j.nanoen.2022.107455

[20] Surmenev RA, Chernozem RV, Pariy IO et al. A review on piezo- and pyroelectric responses of flexible nano- and micropatterned polymer surfaces for biomedical sensing and energy harvesting applications. Nano Energy 2021; 79: 105442. 10.1016/j.nanoen.2020.105442

[21] Chen W, Zheng Q, Lv YA et al. Piezoelectric energy harvesting and dissipating behaviors of polymer-based piezoelectric composites for nanogenerators and dampers. Chem Eng J 2023; 465: 142755. 10.1016/j.cej.2023.142755

[22] Liu D, Yin X, Guo H et al. A constant current triboelectric nanogenerator arising from electrostatic breakdown. Sci Adv 2019; 5: eaav6437. 10.1126/sciadv.aav6437 30972365 PMC6450689

[23] Peng X, Dong K, Ye C et al. A breathable, biodegradable, antibacterial, and self-powered electronic skin based on all-nanofiber triboelectric nanogenerators. Sci Adv 2020; 6: eaba9624. 10.1126/sciadv.aba9624 32637619 PMC7319766

[24] Fu H, Gibson G, Liu Z et al. Piezoelectric wireless power transfer using halbach array for the internet of implanted things. IEEE Internet Things J 2024; 11: 41049–60. 10.1109/JIOT.2024.3457810

[25] Yang Y, Xu L, Jiang D et al. Self-powered controllable transdermal drug delivery system. Adv Funct Mater 2021; 31: 2104092. 10.1002/adfm.202104092

[26] Alagumalai A, Shou W, Mahian O et al. Self-powered sensing systems with learning capability. Joule 2022; 6: 1475–500. 10.1016/j.joule.2022.06.001

[27] Zhao L, Jia S, Fang C et al. Machine learning-assisted wearable triboelectric-electromagnetic sensor for monitoring human motion feature. Chem Eng J 2025; 503: 158637. 10.1016/j.cej.2024.158637

[28] Ma Y, Zheng Q, Liu Y et al. Self-powered, one-stop, and multifunctional implantable triboelectric active sensor for real-time biomedical monitoring. Nano Lett 2016; 16: 6042–51. 10.1021/acs.nanolett.6b01968 27607151

[29] Surmenev RA, Surmeneva MA. The influence of the flexoelectric effect on materials properties with the emphasis on photovoltaic and related applications: a review. Mater Today 2023; 67: 256–98. 10.1016/j.mattod.2023.05.026

[30] Sharmoukh W, Al Kiey SA, Ali BA et al. Recent progress in the development of hole-transport materials to boost the power conversion efficiency of perovskite solar cells. Sustain Mater Technol 2020; 26: e00210. 10.1016/j.susmat.2020.e00210

[31] Wang G, Su Q, Tang H et al. 27.09%-efficiency silicon heterojunction back contact solar cell and going beyond. Nat Commun 2024; 15: 8931. 10.1038/s41467-024-53275-5 39414827 PMC11484749

[32] Helmers H, Höhn O, Lackner D et al. Advancing solar energy conversion efficiency to 47.6% and exploring the spectral versatility of III–V photonic power converters. In: Physics, Simulation, and Photonic Engineering of Photovoltaic Devices XIII. SPIE OPTO, 2024, San Francisco, California, United States. 12881: 1288103.

[33] Razzaq A, Allen TG, Liu W et al. Silicon heterojunction solar cells: Techno-economic assessment and opportunities. Joule 2022; 6: 514–42. 10.1016/j.joule.2022.02.009

[34] Min J, Demchyshyn S, Sempionatto JR et al. An autonomous wearable biosensor powered by a perovskite solar cell. Nat Electron 2023; 6: 630–41. 10.1038/s41928-023-00996-y 38465017 PMC10923186

[35] Lin R, Gao H, Lou J et al. All-perovskite tandem solar cells with dipolar passivation. Nature 2025; 648: 600–6. 10.1038/s41586-025-09773-7 41145173

[36] Huang Z, Li L, Wu T et al. Wearable perovskite solar cells by aligned liquid crystal elastomers. Nat Commun 2023; 14: 1204. 10.1038/s41467-023-36938-7 36864062 PMC9981560

[37] Ren W, Sun Y, Zhao D et al. High-performance wearable thermoelectric generator with self-healing, recycling, and lego-like reconfiguring capabilities. Sci Adv 2021; 7: eabe0586. 10.1126/sciadv.abe0586 33568483 PMC7875524

[38] Kim F, Yang SE, Ju H et al. Direct ink writing of three-dimensional thermoelectric microarchitectures. Nat Electron 2021; 4: 579–87. 10.1038/s41928-021-00622-9

[39] Yang S, Li Y, Deng L et al. Flexible thermoelectric generator and energy management electronics powered by body heat. Microsyst Nanoeng 2023; 9: 106. 10.1038/s41378-023-00583-3 37636323 PMC10449853

[40] Kang M, Yeatman EM. Coupling of piezo- and pyro-electric effects in miniature thermal energy harvesters. Appl Energy 2020; 262: 114496. 10.1016/j.apenergy.2020.114496

[41] Yu B, Wang HQ, Ju L et al. A bio-inspired microwave wireless system for constituting passive and maintenance-free IoT networks. Natl Sci Rev 2024; 12: nwae435. 10.1093/nsr/nwae435 39830403 PMC11737395

[42] Li P, Zhu L, Ding Y et al. Rf energy harvesting for intraoral orthodontic force monitoring. Nano Energy 2024; 121: 109244. 10.1016/j.nanoen.2023.109244

[43] Hu X, Yin W, Du F et al. Biomedical applications and challenges of in-body implantable antenna for implantable medical devices: a review. AEU-Int J Electron Commun 2024; 174: 155053. 10.1016/j.aeue.2023.155053

[44] Kim SH, Basir A, Avila R et al. Strain-invariant stretchable radio-frequency electronics. Nature 2024; 629: 1047–54. 10.1038/s41586-024-07383-3 38778108

[45] Gao S, He T, Zhang Z et al. A motion capturing and energy harvesting hybridized lower-limb system for rehabilitation and sports applications. Adv Sci 2021; 8: 2101834. 10.1002/advs.202101834 PMC8529439 34414697

[46] Lyu S, He Y, Tao X et al. Subcutaneous power supply by NIR-II light. Nat Commun 2022; 13: 6596. 10.1038/s41467-022-34047-5 36329024 PMC9633840

[47] Feng Y, Zhang Z, Gong L et al. Environmental multi-physics coupled tribovoltaic effect for energy harvesting. Natl Sci Open 2025; 4: 20240032. 10.1360/nso/20240032

[48] Gu J, Peng Q, Zhong X et al. Piezo-phototronic PVDF/HfO2/nano-cu heterostructured thin film for flexible self-powered multimodal sensing. Adv Sci 2026; 13: e18913. 10.1002/advs.202518913 PMC12931236 41354624

[49] Kopyl S, Surmenev R, Surmeneva M et al. Magnetoelectric effect: principles and applications in biology and medicine: a review. Mater Today Bio 2021; 12: 100149. 10.1016/j.mtbio.2021.100149 PMC8554634 34746734

[50] Saha O, Truong BD, Roundy S. A review of wireless power transfer using magnetoelectric structures. Smart Mater Struct 2022; 31: 113001. 10.1088/1361-665X/ac9166

[51] Petritz A, Karner-Petritz E, Uemura T et al. Imperceptible energy harvesting device and biomedical sensor based on ultraflexible ferroelectric transducers and organic diodes. Nat Commun 2021; 12: 2399. 10.1038/s41467-021-22663-6 33893292 PMC8065095

[52] Maity K, Garain S, Henkel K et al. Self-powered human-health monitoring through aligned pvdf nanofibers interfaced skin-interactive piezoelectric sensor. ACS Appl Polym Mater 2020; 2: 862–78. 10.1021/acsapm.9b00846

[53] Liu Y, Zhang Q, Huang A et al. Fully inkjet-printed Ag_2_Se flexible thermoelectric devices for sustainable power generation. Nat Commun 2024; 15: 2141. 10.1038/s41467-024-46183-1 38459024 PMC10923913

[54] Du M, Ouyang J, Zhang K. Flexible Bi_2_Te_3_/pedot nanowire sandwich-like films towards high-performance wearable cross-plane thermoelectric generator and temperature sensor array. J Mater Chem A 2023; 11: 16039–48. 10.1039/D3TA02876C

[55] Shaw T, Mandal B, Samanta G et al. Rotation insensitive implantable wireless power transfer system for medical devices using metamaterial-polarization converter. Sci Rep 2024; 14: 19688. 10.1038/s41598-024-70591-4 39181946 PMC11344826

[56] Wang S, Cui Q, Abiri P et al. A self-assembled implantable microtubular pacemaker for wireless cardiac electrotherapy. Sci Adv 2023; 9: eadj0540. 10.1126/sciadv.adj0540 37851816 PMC10584332

[57] Bhatnagar P, Hong J, Patel M et al. Transparent photovoltaic skin for artificial thermoreceptor and nociceptor memory. Nano Energy 2022; 91: 106676. 10.1016/j.nanoen.2021.106676

[58] Xu C, Sun Y, Zhang J et al. Adaptable and wearable thermocell based on stretchable hydrogel for body heat harvesting. Adv Energy Mater 2022; 12: 2201542. 10.1002/aenm.202201542

[59] Liu Z, Xu L, Zheng Q et al. Human motion driven self-powered photodynamic system for long-term autonomous cancer therapy. ACS Nano 2020; 14: 8074–83. 10.1021/acsnano.0c00675 32551540

[60] Chauhan D, Singh AK, Tyagi S et al. Engineering of electrospun lead-free pvdf/carbon nanofiber-zno nanocomposites for enhanced piezoelectric energy harvesting and wearable sensing applications. Compos Part B—Eng 2026; 309: 113039. 10.1016/j.compositesb.2025.113039

[61] Yao H, Xia Z, Wang J et al. Porous, self-polarized ferroelectric polymer films exhibiting behavior reminiscent of morphotropic phase boundary induced by size-dependent interface effect for self-powered sensing. ACS Nano 2024; 18: 9470–85. 10.1021/acsnano.3c11185 38506224

[62] Wu Y, Li Y, Zou Y et al. A multi-mode triboelectric nanogenerator for energy harvesting and biomedical monitoring. Nano Energy 2022; 92: 106715. 10.1016/j.nanoen.2021.106715

[63] Wang T, Shen Y, Chen L et al. Large-scale production of the 3D warp knitted terry fabric triboelectric nanogenerators for motion monitoring and energy harvesting. Nano Energy 2023; 109: 108309. 10.1016/j.nanoen.2023.108309

[64] Zhang Q, Jin T, Cai J et al. Wearable triboelectric sensors enabled gait analysis and waist motion capture for IoT-based smart healthcare applications. Adv Sci 2022; 9: 2103694. 10.1002/advs.202103694 PMC8811828 34796695

[65] Fan C, Long Z, Zhang Y et al. Robust integration of energy harvesting with daytime radiative cooling enables wearing thermal comfort self-powered electronic devices. Nano Energy 2023; 116: 108842. 10.1016/j.nanoen.2023.108842

[66] Waqar A, Sasikumar R, Kim B. Noise-resistant wearable piezoelectric nanogenerator for self-powered energy harvesting and real-time speech-to-text conversion in healthcare monitoring. Nano Energy 2025; 143: 111312. 10.1016/j.nanoen.2025.111312

[67] Lin R, Kim HJ, Achavananthadith S et al. Digitally-embroidered liquid metal electronic textiles for wearable wireless systems. Nat Commun 2022; 13: 2190. 10.1038/s41467-022-29859-4 35449159 PMC9023486

[68] Kim J, Seo J, Jung D et al. Active photonic wireless power transfer into live tissues. Proc Natl Acad Sci USA 2020; 117: 16856–63. 10.1073/pnas.2002201117 32632002 PMC7382277

[69] Rao Y, Xu C, Voss M et al. Fabrication and characterization of a thermoelectric generator with high aspect ratio thermolegs for electrically active implants. Adv Mater Technol 2024; 9: 2301157. 10.1002/admt.202301157

[70] Chung JH, Chowdhury JR, Fan KP et al. Thermoelectric-driven self-powered microneedle sensor for continuous interstitial fluid glucose monitoring. Nano Energy 2025; 146: 111505. 10.1016/j.nanoen.2025.111505

[71] He X, Shi XL, Wu X et al. Three-dimensional flexible thermoelectric fabrics for smart wearables. Nat Commun 2025; 16: 2523. 10.1038/s41467-025-57889-1 40082483 PMC11906656

[72] Xu W, Zhou X, Hao C et al. SLIPS-TENG: robust triboelectric nanogenerator with optical and charge transparency using a slippery interface. Natl Sci Rev 2019; 6: 540–50. 10.1093/nsr/nwz025 34691903 PMC8291521

[73] Niranjan DB, Jacob J, Vaidehi BR et al. Current status and applications of photovoltaic technology in wearable sensors: a review. Front Nanotechnol 2023; 5: 1268931. 10.3389/fnano.2023.1268931

[74] Chen W, Li M, Wang X et al. Flexible Ag_2_Se-based thin-film thermoelectrics for sustainable energy harvesting and cooling. Nat Commun 2025; 16: 7579. 10.1038/s41467-025-62336-2 40813366 PMC12354781

[75] Dagdeviren C, Yang BD, Su Y et al. Conformal piezoelectric energy harvesting and storage from motions of the heart, lung, and diaphragm. Proc Natl Acad Sci USA 2014; 111: 1927–32. 10.1073/pnas.1317233111 24449853 PMC3918766

[76] Khazaee M, Hasani M, Enkeshafi AA et al. Long-term performance of innovative hexa-fold piezoelectric energy harvester for self-powered leadless pacemakers. Smart Mater Struct 2025; 34: 055016. 10.1088/1361-665X/adcfdc

[77] Chung HU, Kim BH, Lee JY et al. Binodal, wireless epidermal electronic systems with in-sensor analytics for neonatal intensive care. Science 2019; 363: eaau0780. 10.1126/science.aau0780 30819934 PMC6510306

[78] Ferreira RG, Silva AP, Nunes-Pereira J. Current on-skin flexible sensors, materials, manufacturing approaches, and study trends for health monitoring: a review. ACS Sens 2024; 9: 1104–33. 10.1021/acssensors.3c02555 38394033 PMC10964246

[79] Zhu P, Mu S, Huang W et al. Soft multifunctional neurological electronic skin through intrinsically stretchable synaptic transistor. Nano Res 2024; 17: 6550–9. 10.1007/s12274-024-6566-8

[80] Jiang Y, Ji S, Sun J et al. A universal interface for plug-and-play assembly of stretchable devices. Nature 2023; 614: 456–62. 10.1038/s41586-022-05579-z 36792740

[81] Chen B, Zhu Y, Yu R et al. Recent progress of biomaterial-based hydrogels for wearable and implantable bioelectronics. Gels 2025; 11: 442. 10.3390/gels11060442 40558741 PMC12192074

[82] Xu H, Zheng W, Zhang Y et al. A fully integrated, standalone stretchable device platform with in-sensor adaptive machine learning for rehabilitation. Nat Commun 2023; 14: 7769. 10.1038/s41467-023-43664-7 38012169 PMC10682047

[83] Zhou H, Jin Z, Xu Y et al. Enhanced laser-induced pedot-based hydrogels for highly conductive bioelectronics. Natl Sci Rev 2025; 12: nwaf136. 10.1093/nsr/nwaf136 40391148 PMC12086669

[84] Chen B, Yu R, Wang J et al. Biomaterials-based hydrogel with superior bio-mimetic ionic conductivity and tissue-matching softness for bioelectronics. Adv Funct Mater 2026; 36: e27495. 10.1002/adfm.202527495

[85] Khatib M, Zhao ET, Wei S et al. High-density soft bioelectronic fibres for multimodal sensing and stimulation. Nature 2025; 645: 656–64. 10.1038/s41586-025-09481-2 40962977

[86] Wang Z, Chen K, Shi X et al. Fibre integrated circuits by a multilayered spiral architecture. Nature 2026; 650: 102–9. 10.1038/s41586-025-09974-0 41565807

[87] Bang J, Choi SH, Pyun KR et al. Bioinspired electronics for intelligent soft robots. Nat Rev Electr Eng 2024; 1: 597–613. 10.1038/s44287-024-00081-2

[88] Long Z, Lin W, Li P et al. One-wire reconfigurable and damage-tolerant sensor matrix inspired by the auditory tonotopy. Sci Adv 2023; 9: eadi6633. 10.1126/sciadv.adi6633 38019910 PMC10686563

[89] Khoshmanesh F, Thurgood P, Pirogova E et al. Wearable sensors: At the frontier of personalised health monitoring, smart prosthetics and assistive technologies. Biosens Bioelectron 2021; 176: 112946. 10.1016/j.bios.2020.112946 33412429

[90] Xu C, Song Y, Sempionatto JR et al. A physicochemical-sensing electronic skin for stress response monitoring. Nat Electron 2024; 7: 168–79. 10.1038/s41928-023-01116-6 38433871 PMC10906959

[91] Zhong B, Qin X, Xu H et al. Interindividual- and blood-correlated sweat phenylalanine multimodal analytical biochips for tracking exercise metabolism. Nat Commun 2024; 15: 624. 10.1038/s41467-024-44751-z 38245507 PMC10799919

[92] Ding S, Saha T, Yin L et al. A fingertip-wearable microgrid system for autonomous energy management and metabolic monitoring. Nat Electron 2024; 7: 788–99. 10.1038/s41928-024-01236-7

[93] Ye C, Wang M, Min J et al. A wearable aptamer nanobiosensor for non-invasive female hormone monitoring. Nat Nanotechnol 2024; 19: 330–7. 10.1038/s41565-023-01513-0 37770648 PMC10954395

[94] Di Giacomo R, Bonanomi L, Costanza V et al. Biomimetic temperature-sensing layer for artificial skins. Sci Robot 2017; 2: eaai9251. 10.1126/scirobotics.aai9251 33157860

[95] Pang Y, Zhang K, Yang Z et al. Epidermis microstructure inspired graphene pressure sensor with random distributed spinosum for high sensitivity and large linearity. ACS Nano 2018; 12: 2346–54. 10.1021/acsnano.7b07613 29378401

[96] You I, Mackanic DG, Matsuhisa N et al. Artificial multimodal receptors based on ion relaxation dynamics. Science 2020; 370: 961–5. 10.1126/science.aba5132 33214277

[97] Covi E, Donati E, Liang X et al. Adaptive extreme edge computing for wearable devices. Front Neurosci 2021; 15: 611300. 10.3389/fnins.2021.611300 34045939 PMC8144334

[98] Matsuhisa N, Niu S O’Neill SJK et al. High-frequency and intrinsically stretchable polymer diodes. Nature 2021; 600: 246–52. 10.1038/s41586-021-04053-6 34880427

[99] Arikpo II, Ogban FU, Eteng IE. Von Neumann architecture and modern computers. Glob J Math Sci 2007; 6: 97–104.

[100] Mondal I, Attri R, Rao TS et al. Recent trends in neuromorphic systems for non-von Neumann in materia computing and cognitive functionalities. Appl Phys Rev 2024; 11: 041304. 10.1063/5.0220628

[101] Ho DH, Roe DG, Choi YY et al. Non-von Neumann multi-input spike signal processing enabled by an artificial synaptic multiplexer. Sci Adv 2022; 8: eabn1838. 10.1126/sciadv.abn1838 35731885 PMC9217087

[102] Xu Z, Zhou T, Ma M et al. Large-scale photonic chiplet Taichi empowers 160-TOPS/W artificial general intelligence. Science 2024; 384: 202–9. 10.1126/science.adl1203 38603505

[103] Jiao Y, Zhao H, Tang J et al. A memristor-based energy-efficient compressed sensing accelerator with hardware-software co-optimization for edge computing. Natl Sci Rev 2026; 13: nwaf499. 10.1093/nsr/nwaf499 41536298 PMC12798729

[104] Mennel L, Symonowicz J, Wachter S et al. Ultrafast machine vision with 2D material neural network image sensors. Nature 2020; 579: 62–6. 10.1038/s41586-020-2038-x 32132692

[105] Chu H, Yan Y, Gan L et al. A neuromorphic processing system with spike-driven SNN processor for wearable ECG classification. IEEE Trans Biomed Circuits Syst 2022; 16: 511–23. 10.1109/TBCAS.2022.3189364 35802543

[106] Wang H, Wang K, Yang J et al. GCN-RL circuit designer: transferable transistor sizing with graph neural networks and reinforcement learning. In: 57th ACM/IEEE Design Automation Conference, San Francisco, CA, 19-22 July 2020.

[107] Prakash K, James A, Valeti NJ et al. Optimization and numerical studies with machine learning assisted graphene-based CuSbS_2_ thin film solar cell for flexible electronics applications. J Phys Chem Solids 2025; 199: 112513. 10.1016/j.jpcs.2024.112513

[108] Hua Q, Khazaee M, Enkeshafi AA et al. Physics-informed surrogate neural network for optimizing diode-based interfaces in leadless pacemaker energy harvesting. IEEE Trans Circuits Syst II, Exp Briefs 2026; 73: 223–7. 10.1109/TCSII.2025.3641701

[109] Ripalda JM, Buencuerpo J, García I. Solar cell designs by maximizing energy production based on machine learning clustering of spectral variations. Nat Commun 2018; 9: 5126. 10.1038/s41467-018-07431-3 30510195 PMC6277435

[110] Bagheri S, Wu N, Filizadeh S. Application of artificial intelligence and evolutionary algorithms in simulation-based optimal design of a piezoelectric energy harvester. Smart Mater Struct 2020; 29: 105004. 10.1088/1361-665X/ab9149

[111] Abbasi Shirsavar M, Taghavimehr M, Ouedraogo LJ et al. Machine learning-assisted E-jet printing for manufacturing of organic flexible electronics. Biosens Bioelectron 2022; 212: 114418. 10.1016/j.bios.2022.114418 35671690

[112] Hussein D, Bhat G, Doppa JR. Adaptive energy management for self-sustainable wearables in mobile health. In: Proceedings of the AAAI Conference on Artificial Intelligence, Palo Alto, CA: AAAI Press, 22 February–1 March 2022.

[113] Geetha BT, Kumar PS, Bama BS et al. Green energy aware and cluster based communication for future load prediction in IoT. Sustainable Energy Technol Assess 2022; 52: 102244. 10.1016/j.seta.2022.102244

[114] Mohamed S, Nomer HAA, Yousri R et al. Energy management for wearable medical devices based on gaining-sharing knowledge algorithm. Complex Intell Syst 2023; 9: 6797–811. 10.1007/s40747-023-01101-8

[115] Maddikunta PKR, Srivastava G, Gadekallu TR et al. Predictive model for battery life in IoT networks. IET Intell Transp Syst 2020; 14: 1388–95. 10.1049/iet-its.2020.0009

[116] Long Z, Li P, Chen J et al. Self-powered single-inductor rectifier-less sshi array interface with the MPPT technique for piezoelectric energy harvesting. IEEE Trans Ind Electron 2022; 69: 10172–81. 10.1109/TIE.2021.3139175

[117] The Cancer Imaging Archive (TCIA) . http://www.cancerimagingarchive.net (11 November 2025, date last accessed).

[118] Fonseca CG, Backhaus M, Bluemke DA et al. The cardiac atlas project—an imaging database for computational modeling and statistical atlases of the heart. Bioinformatics 2011; 27: 2288–95. 10.1093/bioinformatics/btr360 21737439 PMC3150036

[119] Kim KK, Zaluska TJ, Skov S et al. A simplified wearable device powered by a generative emg network for hand-gesture recognition and gait prediction. Nat Sens 2026; 1: 27–38. 10.1038/s44460-025-00002-2

[120] Zhu P, Niu M, Liang S et al. Non-hand-worn, load-free VR hand rehabilitation system assisted by deep learning based on ionic hydrogel. Nano Res 2025; 18: 94907301. 10.26599/NR.2025.94907301

[121] Al-Alim MA, Mubarak R, Salem NM et al. A machine-learning approach for stress detection using wearable sensors in free-living environments. Comput Biol Med 2024; 179: 108918. 10.1016/j.compbiomed.2024.108918 39029434

[122] Bent B, Cho PJ, Henriquez M et al. Engineering digital biomarkers of interstitial glucose from noninvasive smartwatches. Npj Digit Med 2021; 4: 89. 10.1038/s41746-021-00465-w 34079049 PMC8172541

[123] Bozdog IA, Daniel-Nicusor T, Antal M et al. Human behavior and anomaly detection using machine learning and wearable sensors. IEEE 17th International Conference on Intelligent Computer Communication and Processing, Cluj-Napoca, Romania, 2021.

[124] Nasseri M, Pal Attia T, Joseph B et al. Non-invasive wearable seizure detection using long-short-term memory networks with transfer learning. J Neural Eng 2021; 18: 056017. 10.1088/1741-2552/abef8a 33730713

[125] Huang ZX, Liu ZD, Li Y et al. Bpbls: a knowledge-embedded bi-incremental broad learning system for wearable cuffless blood pressure estimation. IEEE J Biomed Health Inform 2026; 30: 5489–502. 10.1109/JBHI.2025.3640660 41348787

[126] Ali H. Reinforcement learning in healthcare: optimizing treatment strategies, dynamic resource allocation, and adaptive clinical decision-making. Int J Comput Appl Technol Res 2022; 11: 88–104. 10.7753/IJCATR1103.1007

[127] Wu X, Li R, He Z et al. A value-based deep reinforcement learning model with human expertise in optimal treatment of sepsis. Npj Digit Med 2023; 6: 15. 10.1038/s41746-023-00755-5 36732666 PMC9894526

[128] Oyeleye M, Chen T, Titarenko S et al. A predictive analysis of heart rates using machine learning techniques. Int J Environ Res Publ Health 2022; 19: 2417. 10.3390/ijerph19042417 PMC8872524 35206603

[129] Walter JR, Lee JY, Yu L et al. Use of artificial intelligence to develop predictive algorithms of cough and PCR-confirmed COVID-19 infections based on inputs from clinical-grade wearable sensors. Sci Rep 2024; 14: 8072. 10.1038/s41598-024-57830-4 38580712 PMC10997665

[130] Hu Q, Chen Y, Xiao J et al. Label-free liver tumor segmentation. IEEE/CVF Conference on Computer Vision and Pattern Recognition (CVPR), Vancouver, IEEE, 2023.

[131] Rahman A, Valanarasu JMJ, Hacihaliloglu I et al. Ambiguous medical image segmentation using diffusion models. IEEE/CVF Conference on Computer Vision and Pattern Recognition (CVPR), Vancouver, IEEE, 2023.

[132] Ye Y, Chang X, Amin F. NE ZHA-TextCNN method for multi-label long text classification. Procedia Comput Sci 2025; 262: 313–9. 10.1016/j.procs.2025.05.058

[133] Ma D, Pang J, Gotway MB et al. A fully open ai foundation model applied to chest radiography. Nature 2025; 643: 488–98.10.1038/s41586-025-09079-840500447

[134] Xiong C, Dang W, Yang Q et al. Integrated ink printing paper based self-powered electrochemical multimodal biosensing (ifp-multi) with chatgpt-bioelectronic interface for personalized healthcare management. Adv Sci 2024; 11: 2305962. 10.1002/advs.202305962 PMC10953564 38161220

[135] Yang H, Li J, Xiao X et al. Topographic design in wearable MXene sensors with in-sensor machine learning for full-body avatar reconstruction. Nat Commun 2022; 13: 5311. 10.1038/s41467-022-33021-5 36085341 PMC9461448

[136] Baccour E, Erbad A, Mohamed A et al. Reinforcement learning-based dynamic pruning for distributed inference via explainable ai in healthcare iot systems. Future Gener Comput Syst 2024; 155: 1–17. 10.1016/j.future.2024.01.021

[137] Zhang Y, Lin X, Yang H et al. A multi-attention feature distillation neural network for lightweight single image super-resolution. Int J Intell Syst 2024; 2024: 3255233. 10.1155/2024/3255233

[138] Asadi M, Poursalim F, Loni M et al. Accurate detection of paroxysmal atrial fibrillation with certified-GAN and neural architecture search. Sci Rep 2023; 13: 11378. 10.1038/s41598-023-38541-8 37452165 PMC10349064

[139] Garifulla M, Shin J, Kim C et al. A case study of quantizing convolutional neural networks for fast disease diagnosis on portable medical devices. Sensors 2022; 22: 219. 10.3390/s22010219 PMC8749713 35009760

[140] Qian L, Lu J, Li W et al. Mcu-enabled epileptic seizure detection system with compressed learning. IEEE Internet Things J 2024; 11: 8771–82. 10.1109/JIOT.2023.3323264

[141] Xie Z, Liu J, Fan J et al. A low-power intelligent ecg monitoring system for wearable devices. IEEE 6th International Conference on Electronics Technology, Chengdu, IEEE, 2023.

[142] Gu M, Zhang Y, Wen Y et al. A lightweight convolutional neural network hardware implementation for wearable heart rate anomaly detection. Comput Biol Med 2023; 155: 106623. 10.1016/j.compbiomed.2023.106623 36809696

[143] Baburaj A, Jayadevan S, Aliyana AK et al. AI-driven TENGS for self-powered smart sensors and intelligent devices. Adv Sci 2025; 12: 2417414. 10.1002/advs.202417414 PMC12120734 40277838

[144] Xiao L, Yin B, Geng Z et al. Flexible wearable devices based on self-powered energy supply. Nano Energy 2025; 142: 111157. 10.1016/j.nanoen.2025.111157

[145] Wang S, Chen X, Zhao C et al. An organic electrochemical transistor for multi-modal sensing, memory and processing. Nat Electron 2023; 6: 281–91. 10.1038/s41928-023-00950-y

[146] Mahajan A, Heydari K, Powell D. Wearable AI to enhance patient safety and clinical decision-making. npj Digit Med 2025; 8: 176. 10.1038/s41746-025-01554-w 40121336 PMC11929813

